# Association of Cardiovascular Outcomes and Mortality With Sustained Long-Acting Insulin Only vs Long-Acting Plus Short-Acting Insulin Treatment

**DOI:** 10.1001/jamanetworkopen.2021.26605

**Published:** 2021-09-24

**Authors:** Emily B. Schroeder, Romain Neugebauer, Kristi Reynolds, Julie A. Schmittdiel, Linda Loes, Wendy Dyer, Noel Pimentel, Jay R. Desai, Gabriela Vazquez-Benitez, P. Michael Ho, Jeffrey P. Anderson, Patrick J. O’Connor

**Affiliations:** 1 Kaiser Permanente Colorado Institute for Health Research, Aurora; 2 Parkview Health, Fort Wayne, Indiana; 3 Division of Research, Kaiser Permanente Northern California, Oakland; 4 Department of Research and Evaluation, Kaiser Permanente Southern California, Pasadena; 5 HealthPartners Institute, Minneapolis, Minnesota; 6 Minnesota Department of Health, St Paul; 7 Rocky Mountain Regional Veterans Affairs and University of Colorado (Anschutz) Medical Center, Denver; 8 HealthPartners Center for Chronic Care Innovation, Minneapolis, Minnesota

## Abstract

**Importance:**

Cardiovascular events and mortality are the principal causes of excess mortality and health care costs for people with type 2 diabetes. No large studies have specifically compared long-acting insulin alone with long-acting plus short-acting insulin with regard to cardiovascular outcomes.

**Objective:**

To compare cardiovascular events and mortality in adults with type 2 diabetes receiving long-acting insulin who do or do not add short-acting insulin.

**Design, Setting, and Participants:**

This retrospective cohort study emulated a randomized experiment in which adults with type 2 diabetes who experienced a qualifying glycated hemoglobin A_1c_ (HbA_1c_) level of 6.8% to 8.5% with long-acting insulin were randomized to continuing treatment with long-acting insulin (LA group) or adding short-acting insulin within 1 year of the qualifying HbA_1c_ level (LA plus SA group). Retrospective data in 4 integrated health care delivery systems from the Health Care Systems Research Network from January 1, 2005, to December 31, 2013, were used. Analysis used inverse probability weighting estimation with Super Learner for propensity score estimation. Analyses took place from April 1, 2018, to June 30, 2019.

**Exposures:**

Long-acting insulin alone or with added short-acting insulin within 1 year from the qualifying HbA_1c_ level.

**Main Outcomes and Measures:**

Mortality, cardiovascular mortality, acute myocardial infarction, stroke, and hospitalization for heart failure.

**Results:**

Among 57 278 individuals (39 279 with data on cardiovascular mortality) with a mean (SD) age of 60.6 (11.5) years, 53.6% men, 43.5% non-Hispanic White individuals, and 4 years of follow-up (median follow-up of 11 [interquartile range, 5-20] calendar quarters), the LA plus SA group was associated with increased all-cause mortality compared with the LA group (hazard ratio, 1.27; 95% CI, 1.05-1.49) and a decreased risk of acute myocardial infarction (hazard ratio, 0.89; 95% CI, 0.81-0.97). Treatment with long-acting plus short-acting insulin was not associated with increased risks of congestive heart failure, stroke, or cardiovascular mortality.

**Conclusions and Relevance:**

Findings of this retrospective cohort study suggested an increased risk of all-cause mortality and a decreased risk of acute myocardial infarction for the LA plus SA group compared with the LA group. Given the lack of an increase in major cardiovascular events or cardiovascular mortality, the increased all-cause mortality with long-acting plus short-acting insulin may be explained by noncardiovascular events or unmeasured confounding.

## Key Points

**Question:**

Among adults with type 2 diabetes receiving long-acting insulin with a glycated hemoglobin A_1c_ level of 6.8% to 8.5%, is there a difference in cardiovascular events and mortality among individuals who do or do not initiate additional treatment with short-acting insulin?

**Findings:**

In this cohort study of 57 278 adults, addition of short-acting insulin was associated with increased all-cause mortality compared with long-acting insulin alone and a decreased risk of acute myocardial infarction, with limited evidence of a difference in congestive heart failure.

**Meaning:**

Given the lack of evidence demonstrating an increase in major cardiovascular events or cardiovascular mortality, the increased mortality with the combination of long- and short-acting insulin may be explained by noncardiovascular events or unmeasured confounding.

## Teaser

This cohort study assesses the occurrence of overall mortality, cardiovascular mortality, acute myocardial infarction, stroke, and hospitalization for congestive heart failure among adults with type 2 diabetes receiving long-acting insulin alone vs long- plus short-acting insulin treatment.

## Introduction

Type 2 diabetes is often a progressive disease. Increasing insulin resistance with decreasing insulin production often necessitates intensification of pharmaceutical therapy over time to maintain control of glucose levels. Often, intensification involves treatment with insulin, with 20% to 30% of adults with type 2 diabetes using insulin at a given time.^1^ For individuals who are already using medication regimens that include basal insulin, the next step is often the addition of prandial, or short-acting, insulin.^2^ Results from trials such as ACCORD (Action to Control Cardiovascular Risk in Diabetes) have raised concerns that, in some circumstances, treatment intensification may lead to increased cardiovascular disease (CVD) or increased overall mortality.^3,4^ This concern is especially important given that major cardiovascular events and cardiovascular mortality are the principal causes of excess mortality and health care costs in adults with type 2 diabetes.^5,6^ However, ACCORD^4^ and similar large randomized trials of glycemic control^7,8^ were not designed to assess the relative effectiveness and safety of specific medications to lower glucose levels.

Starting in 2008, the US Food and Drug Administration has required randomized cardiovascular outcome trials for all new agents to lower glucose levels,^3^ but insulins are specifically exempt from this requirement. Thus, few cardiovascular outcome trials have been completed for long-acting insulins (glargine and degludec),^3,9,10^ and no large randomized studies have compared the cardiovascular outcomes of participants using long-acting insulin alone (LA regimen) with the cardiovascular outcomes of those who are treated with both long-acting and short-acting insulin (LA plus SA regimen). The GRADE (Glycemia Reduction Approaches in Diabetes) study is ongoing and includes approximately 5000 individuals with type 2 diabetes receiving metformin hydrochloride and randomized to additional medications (sulfonylurea, dipeptidyl peptidase 4 inhibitor, glucagonlike peptide 1 receptor agonist, and insulin). However, the primary outcome is time to primary metabolic failure, and it is unlikely to be sufficiently powered to detect an effect on mortality or cardiovascular outcomes.^11^

We therefore conducted a large, multisite retrospective cohort study designed to assess occurrence of mortality, cardiovascular mortality, acute myocardial infarction, stroke (cardiovascular accident [CVA]), and hospitalization for congestive heart failure (CHF) in adults with type 2 diabetes receiving long-acting insulin who did or did not add short-acting insulin after a qualifying hemoglobin A_1c_ (HbA_1c_) level of 6.8% to 8.5% (to convert HbA1c to proportion of total hemoglobin, multiply by 0.01). This HbA_1c_ range was chosen to match that used in the GRADE study^11^ and to represent a range of clinical equipoise in which some individuals may have their diabetes treatment intensified and others would not. The study differs from prior investigations of this topic by including a large number of US participants receiving care in community-based clinics, having relatively complete clinical data and clinical outcome data, and applying current guidelines for modern statistical techniques.

## Methods

### Study Design, Study Sites, and Data Sources

This retrospective cohort study emulated a 4-year randomized experiment^12^ in which adults with previously well-controlled type 2 diabetes who experienced a qualifying HbA_1c_ level of 6.8% to 8.5% while already receiving long-acting insulin would have been randomized at the time of the qualifying HbA_1c_ level to (1) continuing treatment with long-acting insulin alone (LA group) or (2) adding short-acting insulin to long-acting insulin within 1 year (LA plus SA group). The study sites included 4 integrated health care delivery systems from the Health Care Systems Research Network: HealthPartners in Minnesota, Kaiser Permanente Colorado, Kaiser Permanente Northern California, and Kaiser Permanente Southern California.^13^ Health system electronic medical records, administrative claims data, 2010 Census data, and mortality data were used to identify eligible patients, insulin type and use, demographic details, clinical values, outcome variables, and potential covariates. The HealthPartners institutional review board examined, approved, and monitored the progression of this study. The institutional review board approved our request to waive written informed consent for participants owing to the use of retrospective deidentified data. Analysis took place from April 1, 2018, to June 30, 2019. Results and methods are reported in keeping with the Strengthening the Reporting of Observational Studies in Epidemiology (STROBE) guidelines.^14^

### Study Participants

**Figure 1.**
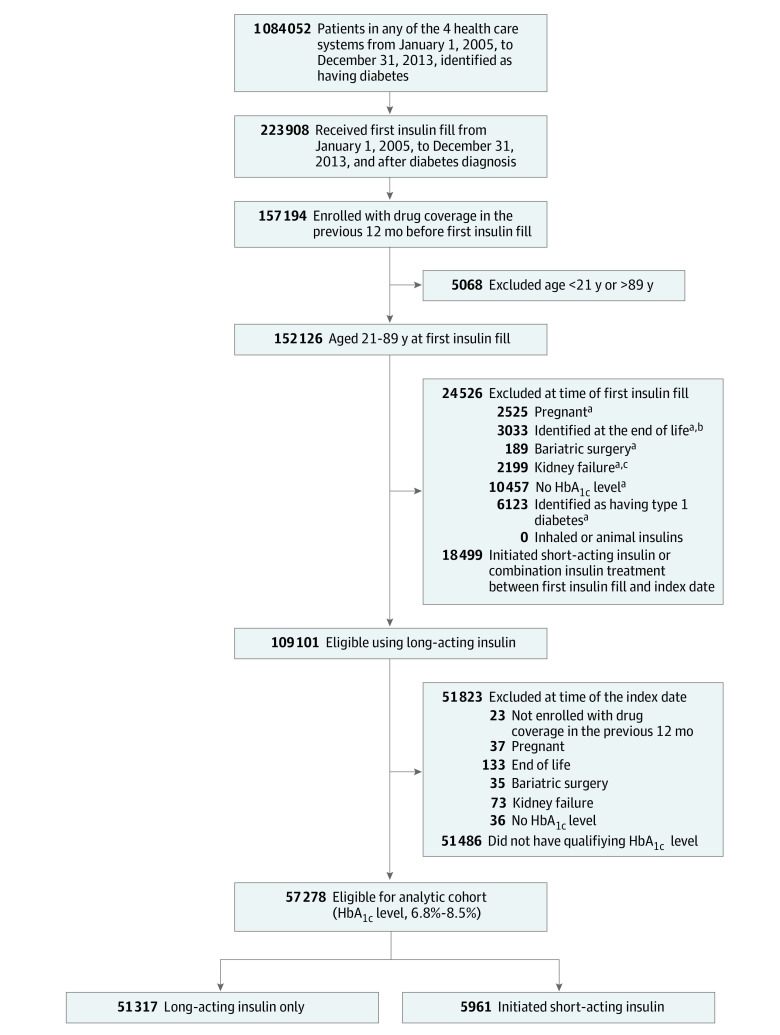
Flowchart Showing Participant Eligibility and Exclusions Data were extracted from the electronic health record and administrative databases using virtual data warehouse databases at each site. Data from January 1, 2005, through December 31, 2013, are included. HbA_1c_ indicates glycated hemoglobin A_1c_. To convert HbA1c to proportion of total hemoglobin, multiply by 0.01. ^a^Identified based on diagnoses, procedure codes, or laboratory test in the 2 years before the index date unless otherwise indicated. ^b^Includes palliative care, hospice care, or stage IV cancer. ^c^Indicates estimated glomerular filtration rate less than 15 mL/min/1.73 m^2^, dialysis, or transplant.

Overall, the combined membership of the 4 participating organizations was approximately 17 million members, of whom approximately 1.1 million individuals met criteria for type 2 diabetes from January 1, 2005, to December 31, 2013 (Figure 1).^15^ As described in eMethods 1 in the Supplement, after identifying adults with diabetes at the 4 participating Health Care Systems Research Network health plans,^15,16^ we applied additional eligibility criteria to limit the analysis to individuals with type 2 diabetes who were recently new users of long-acting insulin and who later experienced a qualifying elevated HbA_1c_ level measurement (that defines the patient’s index date) while receiving long-acting insulin and not short-acting insulin. This process enabled the comparison of individuals receiving long-acting insulin who subsequently either did or did not add short-acting insulin.

Individuals with type 2 diabetes aged 21 to 89 years who had a first initiation of long-acting insulin from January 1, 2005, to December 31, 2013, were potentially eligible. Exclusion criteria consisted of less than 12 months of health plan enrollment and pharmacy coverage before the index date, pregnancy, bariatric surgery in the 2 years before the index date, end-stage kidney disease, evidence of known limited life expectancy (eg, palliative care, hospice, or stage IV cancer), or no HbA_1c_ level measurement in the 2 years before the index date. Individuals could be using noninsulin medications to lower glucose levels before and after the index date.

A qualifying HbA_1c_ level was considered the first HbA_1c_ level measured from 6.8% to 8.5% after the individual had been using long-acting insulin for a minimum of 28 days. This HbA_1c_ range was chosen to match that used in the GRADE study.^11^ The date of the HbA_1c_ measurement was considered the index date, and individuals were considered eligible if they met the above inclusion and exclusion criteria, were still taking long-acting insulin, and had not yet started short-acting insulin therapy on their index date. Further details on the eligibility criteria are provided in eMethods 1 in the Supplement.

Patients were followed up from their index date until their study end date, defined as the earliest of (1) December 31, 2013 (administrative end of the study), (2) plan disenrollment (defined as a health or pharmacy insurance coverage gap >90 days), (3) pregnancy, (4) initiation of a nonstandard insulin therapy (ie, inhaled or animal insulin), or (5) death. For cardiovascular mortality, the administrative end of study was December 31, 2011, owing to a 2-year lag of state death records.

### Exposures

We compared 2 different treatment strategies. In the first treatment strategy, adults with type 2 diabetes receiving long-acting insulin with an HbA_1c_ level from 6.8% to 8.5% continued the treatment with long-acting insulin regardless of subsequent HbA_1c_ levels (LA group). In the second treatment strategy, adults with type 2 diabetes receiving long-acting insulin with an HbA_1c_ level of 6.8% to 8.5% had short-acting insulin added within 1 year of the qualifying HbA_1c_ level (LA plus SA group), with continuous exposure to long-acting insulin before and continuous exposure to both long- and short-acting insulin thereafter. Detailed data on daily insulin dose were not available for analysis.

### Clinical Outcomes

Five clinical time-to-event outcomes were examined (eTable 1 in the Supplement). These outcomes included acute myocardial infarction (*International Classification of Diseases, Ninth Revision, Clinical Modification* [*ICD-9-CM*] code 410.xx), CVA (*ICD-9-CM* codes 430.xx, 431.xx, 433.x1, and 434.x1), and heart failure (*ICD-9-CM* codes 402.01, 402.11, 402.91, 404.01, 404.03, 404.11, 404.13, 404.91, 404.93, and 428.xx) based on the inpatient principal discharge diagnosis. Mortality due to CVD included coronary heart disease, CHF, cerebrovascular disease, peripheral artery disease, and atherosclerosis, as defined by the immediate or primary cause of death.

### Covariates

From our available data sources and based on the current medical literature or consensus medical judgment, the study team (consisting of an experienced cardiologist [P.M.H.], endocrinologist [E.B.S.], primary care physician [P.J.O.], and multiple cardiovascular epidemiologists [K.R., J.A.S., and J.R.D.]) identified a comprehensive list of covariates (eTables 2-6 in the Supplement) potentially associated with the exposures, outcomes, and censoring events (plan disenrollment, adherence to the initial insulin regimen, or death) and including both baseline and time-varying covariates. These covariates included patient demographic details, smoking, laboratory values, vital signs, comorbid conditions, hypoglycemia (defined using *ICD-9-CM* codes), concomitant medications, neighborhood-level socioeconomic variables, and clinician and site characteristics. Race and ethnicity were included as a covariate owing to their potential association with both treatment decisions and outcomes. Race and ethnicity information was collected during routine clinical care, typically by self-report.

### Statistical Analysis

Data were analyzed from April 1, 2018, to June 30, 2019. To emulate a per-protocol analysis^17^ of the comparative effectiveness and safety of the 2 treatment regimens on each outcome, a separate analytic data set was constructed^18^ for each of the 5 clinical outcomes. Measurements on exposure, outcome, censoring, and covariates (eMethods 2 in the Supplement) were updated every 90 days from the index date to the end of follow-up, defined as the earliest of failure occurrence or a right-censoring event. We used the missingness indicator approach to handle partially missing covariate data (eMethods 2 in the Supplement).^19,20,21,22^

To account for both baseline confounding and time-dependent sources of bias from informative censoring,^23^ we used inverse probability weighting (IPW) estimation to evaluate the counterfactual cumulative risks of failure if all patients were continuously exposed to 1 of the 2 insulin treatment regimens described above.^24,25^ More specifically for each outcome, IPW was used to fit 2 logistic marginal structural models (MSMs) for the discrete-time counterfactual hazards (eMethods 3 in the Supplement) during the first 4 years of follow-up: an MSM that relies on the proportionality assumption^26,27^ to provide a single summary effect size measure estimate (hazard ratio [HR]) and a saturated MSM^28,29^ to provide estimates of differences in cumulative risks (at 1, 2, 3, and 4 years) between the 2 exposure regimens without reliance on the proportionality assumption. To minimize parametric assumptions, the first MSM includes a separate term for each quarter of follow-up, which provides the most flexible representation of the baseline hazard function under the proportionality assumption. We also present the *P* value for the test of a difference in the area under the survival curve (AUC) between 2 treatment strategies (eMethods 3 in the Supplement). The AUC difference is also referred to as the restricted mean survival time difference (sum of all the risk differences [RDs] up to a given point in time).^30^ Inferences were derived from prior work^29^ based on the delta method and the influence curve of the IPW estimator of the MSM coefficients.

In the IPW analyses we implemented, person-time observations from the same patient were not assigned to 1 of the 2 treatment regimens as a group at baseline; instead, their assignments to a treatment regimen were determined separately based on the patient’s exposure levels experienced so far, which could result in assignments to both, only 1 of, or none of the 2 treatment regimens evaluated (eMethods 3 in the Supplement). In particular, the same person-time observation could contribute to the evaluation of both counterfactual hazard functions if the exposure history experienced so far by the patient was consistent with a treatment sequence that could be experienced by a trial participant in both arms of the emulated trial (eFigure 1 in the Supplement).

Four approaches for estimating the 11 propensity scores that define the IPW (eMethods 4 in the Supplement) were considered. The first 3 approaches were based on the same general logistic modeling scheme and differed only by the covariate adjustment set^31,32^ that defined each main term of the various propensity score logistic models considered (eMethods 5 and eTables 7-12 in the Supplement). For each of the 5 study outcomes considered, these 3 nested adjustment sets were constructed for estimating each propensity score based on subject matter expertise, going from most restrictive to least restrictive: (1) covariates that affect both failure and the propensity score outcome (ie, either a censoring event or the insulin therapy decision), (2) covariates that are presumed to affect failure, and (3) covariates that affect either failure or the propensity score outcome. The fourth approach was based on data-adaptive propensity score estimation with a machine learning method known as Super Learner.^33^ In this analysis, Super Learner was used to adapt the covariate adjustment set that best estimates each propensity score outcome^34,35^ and to flexibly estimate the propensity scores (eMethods 6 and eTable 13 in the Supplement). For example, the Super Learner approach allows for nonlinear associations when linking continuous variables to propensity scores. All IPWs were stabilized and truncated at 20.^36,37^ Adjusted effect size measure estimates from the 2 MSMs and 4 propensity score estimation approaches considered were also compared with their unadjusted counterparts (ie, derived by fitting the same 2 MSMs without weights). We considered the Super Learner approach to be the primary analysis. Data analyses focused on effect size measures (HR, AUC, and RD) defined through quarter 16 only due to sparse data with longer follow-up periods. Two-sided *P* < .05 indicated statistical significance.

## Results

Of the 1 084 052 patients with type 2 diabetes in the 4 health care systems, 57 278 met study eligibility criteria (Figure 1) and were assessed for the cardiovascular outcomes of interest. Of the 57 278 individuals, only 39 279 could be evaluated in the CVD mortality analyses owing to temporal lags in release of vital statistics mortality data.

Table 1 describes selected demographic and clinical characteristics of the 57 278 patients at the index date in the main cohort by type of insulin treatment initiated. The cohort consisted of 53.6% men, 46.4% women, and 43.5% non-Hispanic White individuals, with a mean (SD) age of 60.6 (11.5) years. The cohort had a high level of comorbidities, with a mean (SD) Elixhauser comorbidity score of 4.7 (2.3), 15.8% prevalence of coronary artery disease, and 5.9% prevalence of CHF. Most individuals were taking noninsulin medications to lower glucose levels, with 66.4% taking metformin and 75.6% taking a sulfonylurea.

**Table 1.** Baseline Characteristics of 57 278 Study Participants by Exposure Group^a^

Variable	Patient group^b^		
	All (N = 57 278)	LA plus SA insulin regimen (n = 5961)	LA insulin regimen (n = 51 317)
Demographic details			
Age, mean (SD), y	60.6 (11.5)	61.0 (11.7)	60.6 (11.4)
Male	30 675 (53.6)	3091 (51.9)	27 584 (53.8)
Female	26 603 (46.4)	2870 (48.1)	23 733 (46.2)
Race and ethnicity			
Asian	6580 (11.5)	646 (10.8)	5934 (11.6)
Black	6016 (10.5)	587 (9.8)	5429 (10.6)
Hawaiian or Pacific Islander	871 (1.5)	84 (1.4)	787 (1.5)
Hispanic	17 010 (29.7)	1509 (25.3)	15 501 (30.2)
Native American	306 (0.5)	35 (0.6)	271 (0.5)
White	24 911 (43.5)	2967 (49.8)	21 944 (42.8)
Missing	1584 (2.8)	133 (2.2)	1451 (2.8)
Tobacco smoking status			
Current	7318 (12.8)	742 (12.4)	6576 (12.8)
Never	28 108 (49.1)	2784 (46.7)	25 324 (49.3)
Former	21 852 (38.2)	2435 (40.8)	19 417 (37.8)
Comorbidities			
Elixhauser comorbidity score, mean (SD)	4.7 (2.3)	5.3 (2.6)	4.7 (2.3)
Hypertension diagnosis^c^	45 308 (79.1)	4836 (81.1)	40 472 (78.9)
CABG surgery or coronary stent^d^	1243 (2.2)	157 (2.76)	1086 (2.1)
Myocardial infarction^e^	750 (1.3)	93 (1.6)	657 (1.3)
CAD^f^	9030 (15.8)	1171 (19.6)	7859 (15.3)
Stroke event^e^	479 (0.8)	73 (1.2)	406 (0.8)
CHF^g^	3380 (5.9)	509 (8.5)	2871 (5.6)
Hospitalization for CHF^e^	641 (1.1)	113 (1.9)	528 (1.0)
Laboratory values and vital signs			
eGFR, mean (SD), mL/min/1.73 m^2^^h^	81.4 (23.2)	76.8 (28.6)	81.9 (28.1)
HbA_1c_ level, mean (SD), %^h^	7.7 (0.5)	7.8 (0.5)	7.6 (0.5)
LDL cholesterol level, mean (SD), mg/dL^h^	83.9 (29.8)	85.2 (31.1)	83.8 (29.6)
Blood pressure, mean (SD), mm Hg^h^			
Systolic	126.0 (14.4)	126.0 (14.9)	126.0 (14.3)
Diastolic	71.5 (9.7)	71.2 (9.9)	71.5 (9.6)
BMI, mean (SD)^i^	32.8 (7.1)	33.3 (7.4)	32.8 (7.0)
Treatment^j^			
Hypertension medications	49 298 (86.1)	5313 (89.1)	43 985 (85.7)
Statins	46 045 (80.4)	4868 (81.7)	41 177 (80.2)
Metformin	38 061 (66.4)	3688 (61.9)	34 373 (67.0)
Sulfonylurea	43 293 (75.6)	4614 (77.4)	38 679 (75.4)
Other diabetes medications	10 238 (17.9)	1242 (20.8)	8996 (17.5)

Abbreviations: BMI, body mass index (calculated as weight in kilograms divided by height in meters squared); CABG, coronary artery bypass graft; CAD, coronary artery disease; CHF, congestive heart failure; eGFR, estimated glomerular filtration rate; HbA1c , glycated hemoglobin A_1c_; IQR, interquartile range; LA, long-acting; LA plus SA, long-acting plus short-acting; LDL, low-density lipoprotein.

SI conversion factor: To convert HbA_1c_ to proportion of total hemoglobin, multiply by 0.01; LDL cholesterol to millimole per liter, multiply by 0.0259.

^a^ Unless otherwise indicated, data are expressed as number (%) of patients. Percentages have been rounded and may not total 100. Variables were assessed at the time of the index date. If laboratory values or vital signs were not available on the index date, the most recent measurement before the index date was used.

^b^ The LA plus SA group includes patients who initiated short-acting insulin therapy in the first year; the LA group includes patients who did not initiate short-acting insulin therapy in the first year and who continued long-acting insulin therapy only or before outcome or censoring events if they occured before the end of the first year.

^c^ From a combination of diagnosis codes, blood pressure, and medications in the prior 2 years.

^d^ From diagnosis codes and procedures codes, ever.

^e^ At least 1 primary inpatient diagnosis code in the prior 2 years.

^f^ At least 2 outpatient diagnosis codes or 1 inpatient diagnosis code in the prior 2 years.

^g^ At least 3 outpatient diagnosis codes or 1 inpatient diagnosis code in the prior 2 years.

^h^ Most recent value in the 2 years before the index date.

^i^ Uses mean weight during the 12 months before the index date.

^j^ Two or more prescriptions in the year before the index date.

In the first year after the index date, 5653 individuals added short-acting insulin while being continually exposed to long-acting insulin from the index date to initiation of short-acting insulin treatment. An additional 5751 individuals added short-acting insulin after the first year and were censored at that point. The median follow-up time was 11 (interquartile range, 5-20) calendar quarters. Most individuals were censored owing to the administrative end of study (77.0%-82.7% depending on outcome), with smaller numbers censored owing to disenrollment from the health plan (12.1%-14.4% depending on outcome) or death (2.8%-5.7% depending on outcome) (eTable 14 in the Supplement).

**Figure 2.**
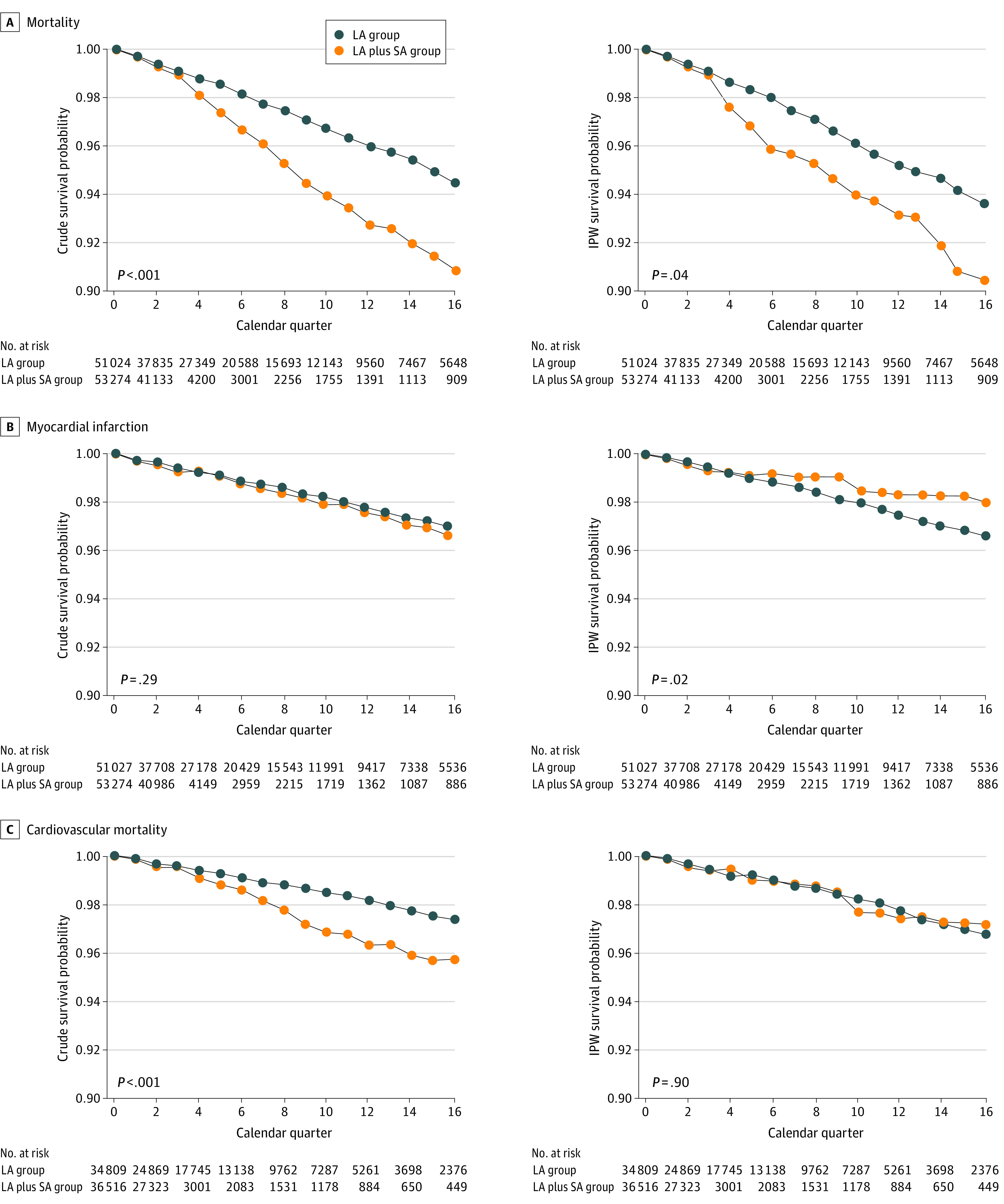
Estimates of the Survival Curves for Overall Mortality, Myocardial Infarction, and Cardiovascular Mortality Outcomes and 2 Insulin Regimens Estimates are derived from a saturated marginal structural model for the counterfactual hazards. The 2 plots in each row display the unadjusted (left) and adjusted (right) estimates of survival probabilities over time and by therapy regimen for each outcome. The *P* value of the statistical test for the area between the 2 survival curves is null (ie, the sum of the risk differences at each quarter is equal to 0) (eFigures 2 and 3 in the Supplement). IPW indicates inverse probability weighting; and LA, long-acting insulin; LA plus SA, long-acting plus short-acting insulin.

**Figure 3.**
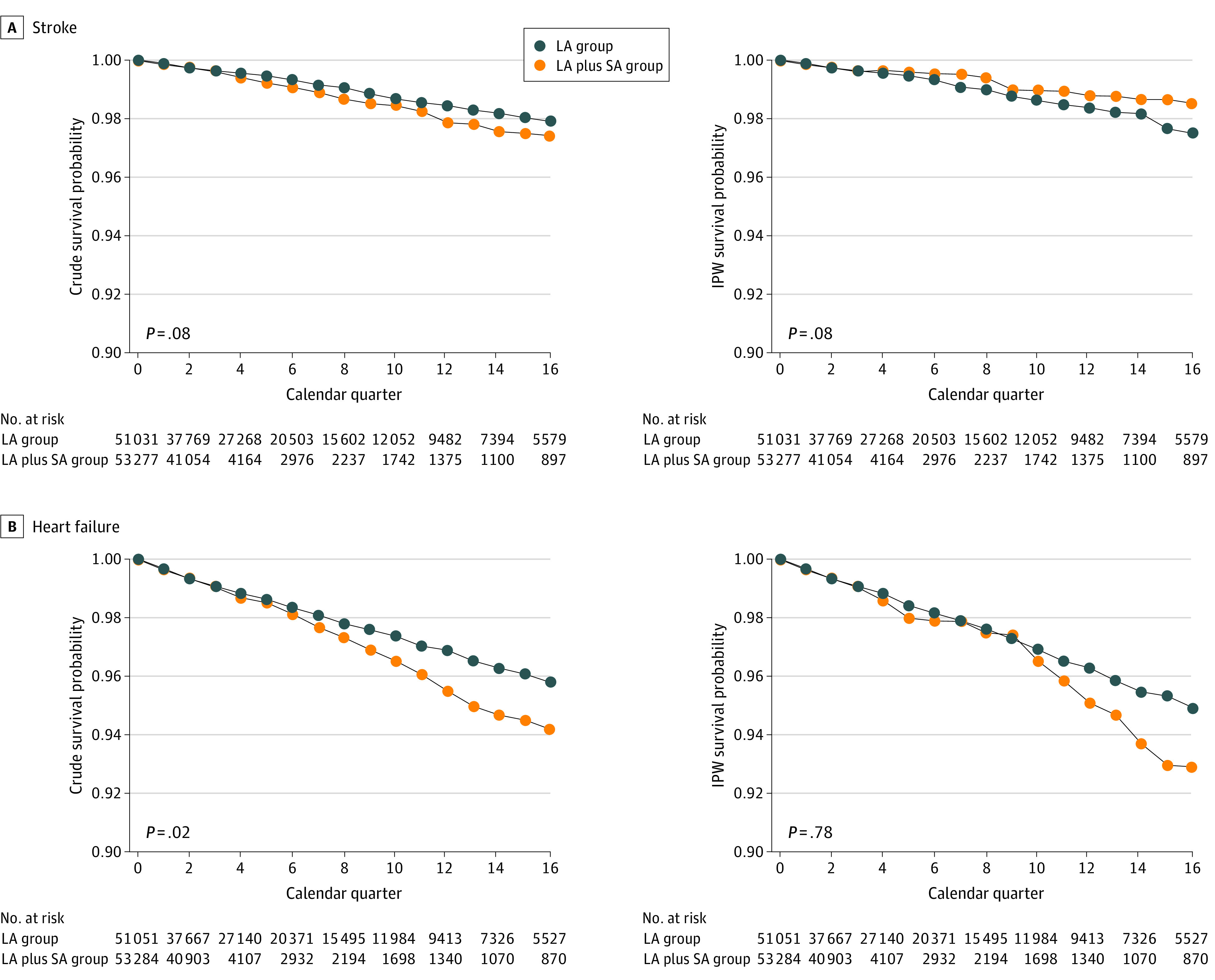
Estimates of the Survival Curves for Stroke and Heart Failure Outcomes and 2 Insulin Regimens Estimates are derived from a saturated marginal structural model for the counterfactual hazards. The 2 plots in each row display the unadjusted (left) and adjusted (right) estimates of survival probabilities over time and by therapy regimen for each outcome. The *P* value of the statistical test for the area between the 2 survival curves is null (ie, the sum of the risk differences at each quarter is equal to 0) (eFigure 2 in the Supplement). IPW indicates inverse probability weighting; LA, long-acting insulin; and LA plus SA, long-acting plus short-acting insulin.

A total of 3612 deaths, 1457 myocardial infarction events, 2040 CHF hospitalizations, 1006 CVA hospitalizations, and 843 deaths due to CVD occurred (eTable 14 in the Supplement). eFigure 1 in the Supplement gives counts of individuals in each exposure group over time. Figure 2 and Figure 3 display the results of the unadjusted (crude) and adjusted (IPW) primary per-protocol analyses based on the saturated MSM and Super Learner for propensity score estimation for each of the 5 outcomes. Table 2 shows the adjusted HRs and RDs with their 95% CIs from the primary per-protocol analyses of each outcome using data-adaptive estimation of the propensity score. Results for the other adjustment approaches are largely consistent with those for the Super Learner approach and are shown in eTables 15 to 20 in the Supplement.

**Table 2.** Point Estimates of HRs and RDs for Each Outcome for the LA Plus SA Insulin Regimen^a^

Outcome	HR (95% CI)	*P* value	AUC	RD (95% CI) at 1 y	*P* value	RD (95% CI) at 2 y	*P* value	RD (95% CI) at 3 y	*P* value	RD (95% CI) at 4 y	*P* value
**Crude**											
Overall mortality	1.41 (1.29 to 1.52)	<.001	0	0.006 (0.004 to 0.009)	<.001	0.022 (0.015 to 0.028)	<.001	0.032 (0.022 to 0.042)	<.001	0.036 (0.023 to 0.050)	<.001
Myocardial infarction	1.04 (0.95 to 1.13)	.35	0.290	−3 × 10^−4^ (−0.002 to 0.001)	.60	0.002 (−0.002 to 0.006)	.23	0.002 (−0.004 to 0.008)	.54	0.004 (−0.005 to 0.012)	.41
Hospitalization for CHF	1.14 (1.06 to 1.22)	.001	0.019	0.002 (0.0003 to 0.004)	.02	0.005 (−0.0003 to 0.010)	.06	0.014 (0.005 to 0.022)	.001	0.016 (0.005 to 0.027)	.003
Stroke (CVA)	1.12 (0.99 to 1.24)	.08	0.078	0.001 (−0.0003 to 0.003)	.13	0.003 (−0.0002 to 0.007)	.07	0.006 (−0.0002 to 0.012)	.06	0.005 (−0.003 to 0.012)	.21
CVD mortality	1.36 (1.16 to 1.57)	<.001	0	0.003 (0.001 to 0.006)	.008	0.010 (0.004 to 0.016)	.001	0.018 (0.009 to 0.028)	<.001	0.017 (0.005 to 0.028)	.004
**Adjusted** ^**b**^											
Overall mortality	1.27 (1.05 to 1.49)	.02	0.037	0.011 (−0.004 to 0.025)	.16	0.018 (0.0001 to 0.037)	.049	0.020 (−0.003 to 0.043)	.09	0.031 (−0.001 to 0.064)	.06
Myocardial infarction	0.89 (0.81 to 0.97)	.005	0.017	−0.0003 (−0.003 to 0.003)	.84	−0.006 (−0.010 to −0.003)	<.001	−0.008 (−0.020 to 0.003)	.15	−0.014 (−0.027 to −0.002)	.03
Hospitalization for CHF	1.07 (0.91 to 1.24)	.40	0.780	0.001 (−0.004 to 0.006)	.63	0.001 (−0.014 to 0.017)	.85	0.012 (−0.012 to 0.037)	.33	0.020 (−0.014 to 0.054)	.24
Stroke (CVA)	0.94 (0.79 to 1.09)	.41	0.078	−0.001 (−0.001 to 0.0003)	.23	−0.004 (−0.007 to −0.002)	.002	−0.004 (−0.013 to 0.005	.34	−0.010 (−0.022 to 0.002)	.10
CVD mortality	1.02 (0.88 to 1.16)	.79	0.900	−0.001 (−0.002 to 0.0001)	.09	−0.001 (−0.008 to 0.005)	.68	0.002 (−0.013 to 0.018)	.76	−0.004 (−0.020 to 0.012)	.63

Abbreviations: AUC, area under the survival curve; CHF, congestive heart failure; CVA, cardiovascular accident; CVD, cardiovascular disease; HR, hazard ratio; LA plus SA, long-acting plus short-acting; RD, risk difference.

^a^ The reference exposure regimen is continuous exposure to long-acting insulin only.

^b^ Inverse probability weighting estimation with Super Learner for propensity score estimation, adjusted for patient demographic details, smoking, laboratory values, vital signs, comorbid conditions, hypoglycemia, concomitant medications, neighborhood-level socioeconomic variables, and clinician and site characteristics (see eMethods 1-7 in the Supplement).

In crude analyses, the LA plus SA regimen was associated with increased HRs and most RDs at years 1 to 4 for overall mortality, CHF, and CVD mortality. For overall mortality, the HR was 1.41 (95% CI, 1.29-1.52), and RDs ranged from 0.006 (95% CI, 0.004-0.009) to 0.036 (95% CI, 0.023-0.050) (an increase of 6-36 events per 1000 persons). For CHF, the HR was 1.14 (95% CI, 1.06-1.22), and the RDs ranged from 0.002 (95% CI, 0.0003-0.004) to 0.016 (95% CI, 0.005-0.027). For CVD mortality, the HR was 1.36 (95% CI, 1.16-1.57), and the RDs ranged from 0.003 (95% CI, 0.001-0.006) to 0.018 (95% CI, 0.009-0.028). For myocardial infarction and stroke, no statistically significant differences were seen, although some results suggested increased risk for the LA plus SA group compared with the LA group.

After adjustment using the Super Learner approach, associations were less consistent. For overall mortality, the adjusted mortality HR was 1.27 (95% CI, 1.05-1.49). The AUC was 0.037, although only the RD at 2 years reached statistical significance (0.018; 95% CI, 0.0001-0.037; *P* = .049), with results suggesting increased risk for the LA plus SA group at the other time points. For myocardial infarction, the HR (0.89 [95% CI, 0.81-0.97]), AUC (0.017), and 2-year RD (−0.006 [95% CI, −0.010 to −0.003]), and 4-year RD (−0.014 [95% CI, −0.027 to −0.002]) showed a lower risk for the LA plus SA compared with the LA regimen. The CHF, stroke, and CVD mortality results were largely nonsignificant, although the 2-year RD for stroke showed a lower risk for the LA plus SA regimen compared with the LA regimen (−0.004 [95% CI, −0.007 to −0.002]).

## Discussion

In this comparison of an LA regimen alone compared with an LA plus SA insulin regimen using a Super Learner strategy with adjustment for patient demographic details, smoking, clinical values, comorbid conditions, concomitant medications, neighborhood-level socioeconomic variables, and clinician and site characteristics, the LA plus SA regimen was associated with increased mortality (HR, 1.27; 95% CI, 1.05-1.49) and lower risk of myocardial infarction (HR, 0.89; 95% CI, 0.81-0.97) compared with the LA regimen alone. The LA plus SA regimen was not associated with significantly different rates of CHF, CVA, and CVD mortality.

The ORIGIN (Outcome Reduction With Initial Glargine Intervention) trial^10^ randomized 12 537 individuals with cardiovascular risk factors plus impaired fasting glucose levels, impaired glucose tolerance, or type 2 diabetes to receive insulin glargine or standard care. No effect on cardiovascular events was found.^10^ Existing studies evaluating insulin strategies and CVD have been limited. The DEVOTE (Trial Comparing Cardiovascular Safety of Insulin Degludec vs Insulin Glargine in Patients With Type 2 Diabetes at High Risk of Cardiovascular Events) trial randomized 7637 individuals with type 2 diabetes to insulin degludec or insulin glargine. More than 85% of the cohort had established CVD, chronic kidney disease, or both. The trial found that insulin degludec was noninferior to insulin glargine with respect to incident major cardiovascular events.^9^ The GRADE study is ongoing and randomized approximately 5000 individuals with type 2 diabetes using metformin to the additional medication regimens (sulfonylurea, dipeptidyl peptidase 4 inhibitor, glucagonlike peptide 1 receptor agonist, and insulin). However, the primary outcome is time to primary metabolic failure, and it is unlikely to be sufficiently powered to detect an effect on cardiovascular outcomes.^11^ We are aware of no other large studies that have specifically compared an LA regimen with an LA plus SA regimen with regard to cardiovascular outcomes.^3^

Hypoglycemia is a common complication of intensive insulin regimens and has been associated with cardiovascular events, cardiovascular mortality, and noncardiovascular mortality.^38,39^ Hypoglycemia rates were consistently higher in the LA plus SA group compared with the LA group, with the largest difference reaching 0.5% at 1 year. Although we were unable to examine hypoglycemia as an outcome, we did adjust for hypoglycemia as a time-varying covariate. However, residual confounding due to unmeasured hypoglycemic events may have remained. In addition, we observed increased overall mortality with the LA plus SA group compared with the LA group but not with increased rates of CVD mortality or cardiovascular events. Owing to our focus on cardiovascular events, we may not have included important confounders of noncardiovascular mortality, which may explain our overall mortality findings. Although our data cannot speak to the existence of specific confounders, important confounders of noncardiovascular mortality that were not included as covariates may include factors such as cancer screening, lifestyle, and important psychosocial factors.

### Strengths and Limitations

Strengths of this study include the large number of US participants receiving care in community-based clinics, having relatively complete clinical data and clinical outcome data and small amounts of loss to follow-up for an observational study, and applying advanced statistical techniques. Although multiple comparisons do not change point estimates and corresponding (pointwise) 95% CIs, there is no correction to our *P* values to compensate for multiple hypothesis testing.

Limitations include the retrospective observational study design, relatively short follow-up periods (median exposure period of 11 calendar quarters), challenges related to measuring the daily dose of insulin and detecting interruption in insulin exposure solely from pharmacy dispensing data, and the potential for unmeasured confounding. In addition, the cohort had a high proportion of individuals using sulfonylureas at baseline (75.6% in the full cohort, with similar proportions in the 2 exposure groups), which was related to formulary structure at the study sites during the period of data collection. Valid causal inferences for per-protocol analyses involving IPW estimation, such as this study, rely on the usual strong assumptions of no unmeasured confounding^25^ or sources of selection bias.^23^ We examined 4 approaches for covariate adjustment, and results from the different approaches were largely consistent. It should also be noted that most of our participants were using human and not analog insulin, which may limit the generalizability of our findings. In addition, our use of the missingness indicator approach based on last observed value carried forward to handle partially missing covariates, such as HbA_1c_ levels, relies on the assumption that treatment decisions are either only affected by the last known covariate measurement (ie, unknown covariate measurements do not affect treatment decisions) or that the effects of unknown covariate measurements on treatment decisions are entirely mediated by other known covariate measurements. If this assumption is violated, then residual (unmeasured) confounding would generally be expected.

## Conclusions

In this retrospective cohort study, we observed an increased risk of all-cause mortality and decreased risk for myocardial infarction for individuals using an LA plus SA insulin regimen compared with those using an LA insulin regimen alone. Given the lack of an increase in major cardiovascular events or cardiovascular mortality, the increased mortality with the LA plus SA insulin regimen may represent noncardiovascular events or unmeasured confounding.

## Notes

Supplement. **eMethods 1.** Cohort Construction **eMethods 2.** Data Structure and Notation **eMethods 3.** Causal Estimands and Inverse Probability Estimator **eMethods 4.** Denominator of the Inverse Probability Weights **eMethods 5.** Standard Propensity Score Estimation With 3 Covariate Adjustment Sets **eMethods 6.** Data-Adaptive Propensity Score Estimation **eMethods 7.** Results **eTable 1.** Sources of Data and Codes Used to Ascertain Major Cardiovascular Events and Mortality **eTable 2.** Part I of II: Brief Description of All Attributes (L) in the Covariate Adjustment Sets **eTable 3.** Part II of II: Brief Description of All Attributes (L) in the Covariate Adjustment Sets **eTable 4.** Part I of II: List of Covariates Considered in the Various Analyses and Whether They Are Assumed to Impact Treatment Decisions, Censoring Events, or Outcomes **eTable 5.** Part II of II: List of Covariates Considered in the Various Analyses and Whether They Are Assumed to Impact Treatment Decisions, Censoring Events, or Outcomes **eTable 6.** Cutoffs Used to Discretize Continuous Covariates **eTable 7.** Propensity Score Estimation Approach 1 in the AMI Analysis **eTable 8.** Propensity Score Estimation Approach 2 in AMI Analysis (Part I of II) **eTable 9.** Propensity Score Estimation Approach 2 in AMI Analysis (Part II of II) **eTable 10.** Propensity Score Estimation Approach 3 in AMI Analysis (Part I of III) **eTable 11.** Propensity Score Estimation Approach 3 in AMI Analysis (Part II of III) **eTable 12.** Propensity Score Estimation Approach 3 in AMI Analysis (Part III of III) **eTable 13.** Propensity Score Estimation Approach 4 in AMI Analysis **eTable 14.** Event Rates and Reasons for End of Analytic Follow-up **eFigure 1.** Data Support in the AMI (Top Panel) and CVD Mortality (Bottom Panel) Analyses **eTable 15.** AMI Results **eTable 16.** CHF Results **eTable 17.** CVA Results **eTable 18.** CVD Mortality Results **eTable 19.** All-Cause Mortality Results **eFigure 2.** Survival Curve Estimates for AMI, CHF, and CVA Based on the Saturated MSM **eFigure 3.** Survival Curve Estimates for CVD and All-Cause Mortality Based on the Saturated MSM **eTable 20.** Summary Statistics of the Inverse Probability Weights (IPW)
eReferences.
