# Supplementary material for: Association of Cardiovascular Outcomes and Mortality With Sustained Long-Acting Insulin Only vs Long-Acting Plus Short-Acting Insulin Treatment
Source: JAMA Netw Open. 2021 Sep 24;4(9):e2126605. doi: 10.1001/jamanetworkopen.2021.26605 (PMC8463942; doi:10.1001/jamanetworkopen.2021.26605)
Supplement: Supplement. — eMethods 1. Cohort Construction eMethods 2. Data Structure and Notation eMethods 3. Causal Estimands and Inverse Probability Estimator eMethods 4. Denominator of the Inverse Probability Weights eMethods 5. Standard Propensity Score Estimation With 3 Covariate Adjustment Sets eMethods 6. Data-Adaptive Propensity Score Estimation eMethods 7. Results eTable 1. Sources of Data and Codes Used to Ascertain Major Cardiovascular Events and Mortality eTable 2. Part I of II: Brief Description of All Attributes (L) in the Covariate Adjustment Sets eTable 3. Part II of II: Brief Description of All Attributes (L) in the Covariate Adjustment Sets eTable 4. Part I of II: List of Covariates Considered in the Various Analyses and Whether They Are Assumed to Impact Treatment Decisions, Censoring Events, or Outcomes eTable 5. Part II of II: List of Covariates Considered in the Various Analyses and Whether They Are Assumed to Impact Treatment Decisions, Censoring Events, or Outcomes eTable 6. Cutoffs Used to Discretize Continuous Covariates eTable 7. Propensity Score Estimation Approach 1 in the AMI Analysis eTable 8. Propensity Score Estimation Approach 2 in AMI Analysis (Part I of II) eTable 9. Propensity Score Estimation Approach 2 in AMI Analysis (Part II of II) eTable 10. Propensity Score Estimation Approach 3 in AMI Analysis (Part I of III) eTable 11. Propensity Score Estimation Approach 3 in AMI Analysis (Part II of III) eTable 12. Propensity Score Estimation Approach 3 in AMI Analysis (Part III of III) eTable 13. Propensity Score Estimation Approach 4 in AMI Analysis eTable 14. Event Rates and Reasons for End of Analytic Follow-up eFigure 1. Data Support in the AMI (Top Panel) and CVD Mortality (Bottom Panel) Analyses eTable 15. AMI Results eTable 16. CHF Results eTable 17. CVA Results eTable 18. CVD Mortality Results eTable 19. All-Cause Mortality Results eFigure 2. Survival Curve Estimates for AMI, CHF, and CVA Based on the Saturated MSM eFigure 3. Survival Curve Estimates fo [file jamanetwopen-e2126605-s001.pdf]

Supplementary Online Content

Schroeder EB, Neugebauer R, Reynolds K, et al. Association of cardiovascular outcomes and mortality with sustained long-acting insulin only vs long-acting plus short-acting insulin treatment. *JAMA Netw Open*. 2021;4(9):e2126605. doi:10.1001/jamanetworkopen.2021.26605

eMethods 1. Cohort Construction
eMethods 2. Data Structure and Notation
eMethods 3. Causal Estimands and Inverse Probability Estimator
eMethods 4. Denominator of the Inverse Probability Weights
eMethods 5. Standard Propensity Score Estimation With 3 Covariate Adjustment Sets
eMethods 6. Data-Adaptive Propensity Score Estimation
eMethods 7. Results
eTable 1. Sources of Data and Codes Used to Ascertain Major Cardiovascular Events and Mortality
eTable 2. Part I of II: Brief Description of All Attributes (L) in the Covariate Adjustment Sets
eTable 3. Part II of II: Brief Description of All Attributes (L) in the Covariate Adjustment Sets
eTable 4. Part I of II: List of Covariates Considered in the Various Analyses and Whether They Are Assumed to Impact Treatment Decisions, Censoring Events, or Outcomes
eTable 5. Part II of II: List of Covariates Considered in the Various Analyses and Whether They Are Assumed to Impact Treatment Decisions, Censoring Events, or Outcomes
eTable 6. Cutoffs Used to Discretize Continuous Covariates
eTable 7. Propensity Score Estimation Approach 1 in the AMI Analysis
eTable 8. Propensity Score Estimation Approach 2 in AMI Analysis (Part I of II)
eTable 9. Propensity Score Estimation Approach 2 in AMI Analysis (Part II of II)
eTable 10. Propensity Score Estimation Approach 3 in AMI Analysis (Part I of III)
eTable 11. Propensity Score Estimation Approach 3 in AMI Analysis (Part II of III)
eTable 12. Propensity Score Estimation Approach 3 in AMI Analysis (Part III of III)
eTable 13. Propensity Score Estimation Approach 4 in AMI Analysis
eTable 14. Event Rates and Reasons for End of Analytic Follow-up
eFigure 1. Data Support in the AMI (Top Panel) and CVD Mortality (Bottom Panel) Analyses
eTable 15. AMI Results
eTable 16. CHF Results
eTable 17. CVA Results
eTable 18. CVD Mortality Results
eTable 19. All-Cause Mortality Results
eFigure 2. Survival Curve Estimates for AMI, CHF, and CVA Based on the Saturated MSM
eFigure 3. Survival Curve Estimates for CVD and All-Cause Mortality Based on the Saturated MSM
eTable 20. Summary Statistics of the Inverse Probability Weights (IPW)
eReferences.

This supplementary material has been provided by the authors to give readers additional information about their work.

©2021 Shroeder EB et al. *JAMA Network Open*.

eMethods 1 - Cohort Construction

We searched the electronic health records and administrative databases of the HP, KPCO, KPNC, KPSC health plans to identify all diabetes patients between 1/1/2000 and 12/31/2013 with a first insulin dispensing composed of only long-acting insulin between 1/1/2005 and 12/31/2013 who had a first elevated A1c between 6.8% and 8.5% 28 days or more after initiation of long-acting insulin. The algorithm used to identify diabetes patients is described below (second bullet). The elevated A1c date is referred to as the index date. Each patient who met all of the following criteria was included in the main study cohort:

- age on date of first long-acting insulin dispensing and index date $\geq$21 and $\leq$89
- diabetes recognition occurred before or on first long-acting insulin dispensing where the diabetes recognition date was defined from the patient's diagnoses from inpatient, ambulatory, laboratory, and pharmacy encounters. Specifically, diabetes recognition was defined as the earlier of one inpatient diagnosis (ICD-9-CM 250.x, 357.2, 366.41, 362.01-362.07) or any combination of two of the following events occurring within a 24-month period of time, using the date of the first event in the pair as the identification date: 1) A1C > 6.5% (48 mmol/mol); 2) fasting plasma glucose > 126 mg/dl (7.0 mmol/L); 3) random plasma glucose > 200 mg/dl (11.1 mmol/L); 4) an outpatient diagnosis code (same codes as inpatient); 5) any anti-hyperglycemic medication dispensed. For example, an individual with an A1C of 7.5% (57 mmol/mol) followed by an outpatient diagnosis of diabetes would be identified with diabetes on the (earlier) date of the A1C, with a laboratory result as the primary source. When the two events used for identification came from the same source (e.g., two outpatient diagnoses), they were required to occur on separate dates, but no more than 24-months apart. Note the following exception: two dispensings of metformin, thiazolidinediones, or liraglutide – with no other indication of diabetes – was not counted because these agents could be used for diabetes prevention, weight loss or to treat polycystic ovarian syndrome. Events that were identified during a pregnancy (within 270 days prior to a delivery) were excluded from consideration
- the elevated A1c on the index date occurred while on long-acting insulin and no short-acting insulin was filled between the first long-acting dispensing and the index date
- minimum of 12 months of health plan enrollment before first long-acting insulin dispensing and allowing for multiple gaps not exceeding 90 days combined. This criterion was also required for the 12 months before index date.
- minimum of 12 months of drug coverage before first long-acting insulin dispensing and allowing for multiple gaps not exceeding 90 days combined. This criterion was also required for the 12 months before index date.
- not pregnant on first long-acting insulin dispensing and on index date
- no evidence of bariatric surgery in the 2 years before first long-acting insulin dispensing, i.e., no record of the following ICD-9 procedure and CPT-4 codes: 43.89, 44.31, 44.38, 44.39, 44.68, 44.69, 44.95 ; 43633, 43644, 43645, 43659, 43770, 43775, 43842, 43843, 43844, 43845, 43846, 43847. This criterion was also required in the 2 years before index date.
- no evidence of end stage renal disease in the 2 years before first long-acting insulin dispensing, i.e., no record of the following ICD-9 diagnosis, ICD-9 procedure, and CPT-4 codes (kidney transplant): v42.0, 996.81 ; 55.6, 55.61, 55.69 ; 50360, 50365, 50380 and most recent GFR laboratory result (if any)$\geq$15 and no record of 2 or more of the following ICD-9 diagnosis, ICD-9 procedure, and CPT-4 codes dated >90 days apart as primary or secondary diagnosis (dialysis): 585.6, 458.21, v45.1, v45.11, v56, v56.x, v56.2, v56.8 ; 39.95, 54.98 ; 90921, 90925, 90935-90999. This criterion was also required in the 2 years before index date.
- no evidence of a stage 4 cancer diagnosis in the 2 years before first long-acting insulin dispensing, i.e., no record of the following ICD-9 diagnosis codes 197.x, 198.x, 199.x. This criterion was also required in the 2 years before index date.

©2021 Shroeder EB et al. *JAMA Network Open*.

- no evidence of hospice or palliative care in the 2 years before first long-acting insulin dispensing, i.e., no record of an hospice encounter and no record of the ICD-9 diagnosis code v66.7 and no record of the CPT code 99377 and 99378. This criterion was also required in the 2 years before index date.

- at least one A1c laboratory measurement recorded in the 2 years before first long-acting insulin dispensing. This criterion was also required in the 2 years before index date.

- insulins dispensed on first long-acting insulin dispensing do not include animal, inhaled, or short-acting insulins

- diabetes of type 2 defined by the following ratio being strictly lower than 50%: the number of ICD-9 diagnosis codes 250.x1 and 250.x3 (type 1) in the 2 years before first long-acting insulin dispensing divided by the sum of this number and the number of ICD-9 codes 250.x0 and 250.x2 (type 2) in the 2 years before first long-acting insulin dispensing. If this ratio is not defined (i.e., denominator is 0), the diabetes type is unknown and the patient excluded from the study cohort. This criterion was also required in the 2 years before index date.

In addition to these criteria above, KPCO patients living outside the Denver/Boulder area were excluded due to incomplete data capture.

eMethods 2 - Data Structure and Notation

All analyses in this report are based on analytic datasets constructed with the MSMstructure SAS macro[1] to coarsen daily EHR data using the 90-day unit of time, i.e., time-dependent variables are updated every 90 days in the resulting analytic datasets. More specifically, for each of the five failure time outcomes considered (eTable 1), a separate analytic dataset is constructed by collecting the realizations of the random variables described below for all patients in the main or CVD study cohort.

Follow-up time (expressed in 90-day units) is denoted by t and, by convention, the first 90 days of follow-up are denoted by $t = 0$. The time when the patient's follow-up ends is denoted by $\tilde{T}$ and is defined as the earliest of the time to failure denoted by T or the time to a right-censoring event denoted by C. When a patient is right-censored, i.e., $C < T$, the type of right-censoring event experienced by the patient is recorded and denoted by Γ with possible values 1-5 to represent the administrative end of study, disenrollment from the health plan, start of a pregnancy, initiation of a non standard insulin (i.e., inhaled or animal insulin), or death, respectively. The indicator that the end of follow-up is due to the occurrence of a failure event is denoted by $\Delta = I(T \leq C)$, i.e., $\Delta = 1$ implies that $\tilde{T} = T$ and $\Delta = 0$ implies that $\tilde{T} = C$. Treatment with insulin therapy at time t is represented by the categorical variable $A_1(t)$ with four possible levels: long-acting insulin only (encoded by 0), both long-acting and short-acting insulin (encoded by 1), no insulin therapy (encoded by 2), short-acting insulin only (encoded by 3). The indicator of the patient's right-censored status at time t is denoted by $A_2(t)$. We thus have $A_2(t) = 0$ for $t = 0, \ldots, \tilde{T} - 1$ when $\tilde{T} \geq 1$ and $A_2(\tilde{T}) = 1 - \Delta$. The exposure variable denoted by $A(t)$ is defined by $A(t) = (A_1(1), A_2(t))$. At each time point $t = 0, \ldots, \tilde{T}$, covariates such as A1c measurements (eTables 2-3) are denoted by a component $L_j(t)$ of the random vector $L(t)$ and defined from measurements that occur before the exposure at time t, $A(t)$, or are otherwise assumed not to be affected by the exposures at time t or thereafter, $(A(t), A(t + 1), \ldots)$. If no such measurements were collected, each variable $L_j(t)$ is defined by convention using last observed value carried forward at $t > 0$. If no baseline measurements were collected for a continuous variable in $L(0)$, the variable is defined by convention as the median of the baseline values from patients with observed measurements at $t = 0$. For categorical variables in $L(0)$, a separate level is defined to encode missing baseline measurements. For each time-independent or time-dependent covariate L_j with at least one missing measurement (at baseline or at $t > 0$), an indicator of missing covariate measurement at time t is created and included as a distinct variable (e.g., to encode intensity of clinical monitoring) in the random vector $L(t)$ for all time points t. In addition, the vector of covariates $L(t)$ at time t include an outcome measurement denoted by $Y(t)$, i.e., $Y(t) \in L(t)$ for $t = 0, \ldots, \tilde{T}$. For each time point $t = 1, \ldots, \tilde{T} + 1$, the outcome is the indicator of past failure, i.e., $Y(t) = I(T \leq t - 1)$ and $Y(0) = 0$ by convention. By definition, the outcome is thus 0 for $t = 0, \ldots, \tilde{T}$, not observed at $t = \tilde{T} + 1$ if $\Delta = 0$ and, 1 at $t = \tilde{T} + 1$ if $\Delta = 1$.

In short, the observed data in each analytic dataset are realizations of n copies O_i of the random process $O = \tilde{T}, \Delta, (1 - \Delta)\Gamma, \bar{L}(\tilde{T}), \bar{A}(\tilde{T}), \Delta Y(\tilde{T} + 1)$ where $n = 57,278$ in each of the four analytic datasets to evaluate AMI, CHF, CVA, and all-cause mortality, and $n = 39,279$ in the analytic dataset to evaluate CVD mortality. In the analyses of each dataset, we assumed[2] that the random variables O_i are independent and identically distributed and we denote their common distribution with P_0.

To simplify expressions below, we use the overbar notation ¯ to denote the history of a variable from baseline to time t (e.g., $\bar{A}(t) = (A(0), \ldots, A(t))$) and, by convention, $L(t)$ and $A(t)$ are nil when $t < 0$. Lower case notation is used to represent a possible realization of a random variable.

eMethods 3 - Causal Estimands and Inverse Probability Estimator

The causal estimands of interest are defined by two exposure regimens over the first 16 quarters of follow-up: 1) a static regimen denoted by g_0^* and defined by continuous exposure to only long-acting insulin without occurrence of right-censoring events, and 2) a stochastic exposure regimen denoted by g_1^* and defined by continuous exposure to long-acting and short-acting insulin without occurrence of right-censoring events where patients are given a 4-quarter grace period[3] after the index date to intensify insulin therapy through the addition of short-acting insulin. Formally, both interventions can be defined as stochastic interventions, i.e., the collection of conditional probabilities [4, Section 6], as follows.

Continuous insulin therapy with only long-acting insulin:

$$g_0^*\left(a(t) = (0,0) \mid \bar{l}(t), y(t) = 0, a_2(t-1) = 0, \bar{a}(t-1)\right) = 1 \text{ for all } t = 0, \ldots, 15$$

This stochastic intervention implies the following joint static intervention: $\bar{a}_1(15) = (0, \ldots, 0)$ (continuous treatment with only long-acting insulin) and $\bar{a}_2(15) = (0, \ldots, 0)$ (no right-censoring events).

Continuous insulin therapy with long-acting insulin and treatment intensification in the first 4 quarters of follow-up through the addition of short-acting insulin and continuous exposure to both long- and short-acting insulin thereafter:

- no right-censoring events

$$g_1^*\left(a_2(t) = 0 \mid \bar{l}(t), y(t) = 0, a_2(t-1) = 0, \bar{a}(t-1)\right) = 1 \text{ for all } t = 0, \ldots, 15$$

- patient is free to initiate short-acting insulin at any time before the last quarter of the grace period with probabilities that approximate patterns of treatment intensification observed in the cohort

$$g_1^*\left(a_1(t) = 1 \mid \bar{l}(t), y(t) = 0, a_2(t-1) = 0, \bar{a}(t-1), a_1(t-1) = 0\right) = 1 - g_0(a_1(t) = 0 \mid y(t) = 0, \bar{a}_2(t) = 0, \bar{a}_1(t-1) = 0, a_1(t) \in \{0;1\}) \text{ for all } t \leq 2$$

where the probabilities g_0 are defined by the distribution of the observed data process P_0. These probabilities were estimated using the sample mean and were equal to ≈ 0.97 at quarters 0, 1, and 2. They represent the proportions of patients in the real data that remained treated with only long-acting insulin at quarter t among patients who were previously treated with only long-acting insulin and who were either treated with only long-acting insulin or both long-acting or short-acting insulin at quarter t. With this intervention definition $\approx$ 8.7% of patients would on average initiate short-acting insulin therapy at quarter 0, 1, or 2 in the arm of an ideal randomized experiment if patients perfectly complied with the assigned intervention g_1^*. This can be contrasted with the rate of treatment intensification with short-acting insulin in the observed data used, for example, to examine AMI: 8.3% (4,729) of the $n = 57,278$ patients initiated short-acting insulin therapy in quarter 0,1, or 2.

- once short-acting insulin is initiated, patient must continue use of both insulin types

$$g_1^*\left(a_1(t) = 1 \mid \bar{l}(t), y(t) = 0, a_2(t) = 0, \bar{a}(t-1), a_1(t-1) = 1\right) = 1 \text{ for all } t \leq 2$$

- patient must use both long- and short-acting insulin during and after the third quarter of follow-up

$$g_1^*\left(a_1(t) = 1 \mid \bar{l}(t), y(t) = 0, a_2(t) = 0, \bar{a}(t-1)\right) = 1 \text{ for all } t \geq 3.$$

The stochastic intervention g_1^* implies the following joint interventions: $\bar{a}_2(15) = (0, \ldots, 0)$ (no right-censoring events) and $\bar{a}_1(15) = (0, \ldots, 0, 1, \ldots, 1)$ where treatment intensification with short-acting insulin occurs during the first 4 quarters of follow-up.

©2021 Shroeder EB et al. *JAMA Network Open*.

The following two working,[5] logistic, marginal structural models (MSMs) for the discrete-time counterfactual hazards defined by the prior two stochastic interventions, $P(Y_{g_x^*}(t+1) = 1 \mid Y_{g_x^*}(t) = 0)$ with $x = 0, 1$, were considered:

- a simple working MSM whose parameterization mimics a common modeling practice that assumes constant hazard ratios over time (i.e., a model based on the proportionality assumption):

$$m_1(t, x \mid \beta) = \left(1 + \exp\left(-\left(\beta^0 x + \sum_{j=1}^{16} \beta^j I\left(t = j-1\right)\right)\right)\right)^{-1}$$

- a saturated working MSM whose parameterization permits hazard ratios to change over time:

$$m_2(t, x \mid \beta) = \left(1 + \exp\left(-\sum_{j=1}^{16}\sum_{k=0}^{1} \beta^{j,k} I\left(t = j-1, x = k\right)\right)\right)^{-1}$$

for $t = 0, \ldots, 15$ where, for each working MSM, the collection of its coefficients is denoted by β.

A standard[4,6,7] bounded and stabilized IPW estimator approach was implemented to fit each working MSM through a weighted logistic regression where each person-time-observation $(A(t), Y(t+1))$ for $t = 0, \ldots, 15$ was duplicated for each regimen $x = 0, 1$ and assigned the following inverse probability weight:

$$\frac{\lambda(x) \prod_{j=0}^{t} g_x^*(A(j) \mid \bar{L}(j), \bar{A}(j-1))}{\prod_{j=0}^{t} g_0(A(j) \mid \bar{L}(j), \bar{A}(j-1))}, \tag{1}$$

with the following choice of stabilizing factor $\lambda(x) = \prod_{j=0}^{t} P_n\left(F_x(j) = 1 \mid \bar{F}_x(j-1) = 1\right)$ where each factor P_n denotes a sample mean and $F_x(j)$ is defined as the indicator that the patient followed the intervention g_x^* at time j, i.e., $F_x(j) = I\left(g_x^*(A(j) \mid \bar{L}(j), \bar{A}(j-1)) > 0\right)$. The resulting IPW estimator of the working MSM coefficient β is denoted by β_n and define the various effect measures reported below.

The first MSM fit provided a single effect measure estimate, $\exp(\beta_n^0)$, corresponding with an estimate of the constant causal hazard ratio (HR) $(P(Y_{g_1^*}(t+1) = 1 \mid Y_{g_1^*}(t+1) = 0)/P(Y_{g_0^*}(t+1) = 1 \mid Y_{g_0^*}(t+1) = 0))$ under the proportionality and rare event assumptions. The second MSM fit was mapped into estimates of the counterfactual cumulative risks $P(Y_{g_x^*}(t+1) = 1)$ (equivalently, the counterfactual survival probability $P(T_{g_x^*} > t) = 1 - P(Y_{g_x^*}(t+1) = 1)$) as follows for $t = 0, \ldots, 15$ and $x = 0, 1$:

$$P_n(Y_{g_x^*}(t+1) = 1) = 1 - \prod_{j=0}^{t}\left(1 - m_2(j, x \mid \beta_n)\right).$$

These estimates of counterfactual cumulative risks defined the following additional effect measure estimates:

- the difference between the areas under the two discrete-time survival curves (AUC):

$$\sum_{j=0}^{15} P_n(Y_{g_1^*}(j+1) = 1) - P_n(Y_{g_0^*}(j+1) = 1)$$

- the risk difference (RD) at year $j = 1, \ldots, 4$: $P_n(Y_{g_1^*}(4 * j) = 1) - P_n(Y_{g_0^*}(4 * j) = 1)$

Inferences for the AUC and RD effect measures were derived from prior work[7] based on the delta method and the influence curve of the IPW estimator β_n.

eMethods 4 - Denominator of the Inverse Probability Weights

The denominators of the IP weights (1) used to fit each of the two working MSM described above require estimation of the conditional probabilities $g_0(A(t) = a^x(t) \mid \bar{L}(t), \bar{Y}(t) = 0, \bar{A}(t-1) = \bar{a}^x(t-1))$ for $t = 0, \ldots, 15$ when $\prod_{j=0}^{t} g_x^*(A(j) = a^x(j) \mid \bar{L}(j), \bar{A}(j-1) = \bar{a}^x(j-1)) > 0$ with $x = 0$ or 1. These probabilities can be factorized based on the following 11 propensity scores (PS) for:

- continuation of only long-acting insulin therapy in the first quarter of follow-up among patients not right-censored in the first quarter (PS denoted by $\mu_1(0)$):

$$g_0 \;\; A_1(0) = 0 \;\; L(0), A_2(0) = 0$$

- initiation of short-acting insulin therapy and continued use of long-acting insulin in the first quarter of follow-up among patients not right-censored in the first quarter and who interrupted treatment with only long-acting insulin during quarter 1 (PS denoted by $\mu_2(0)$):

$$g_0 \;\; A_1(0) = 1 \;\; L(0), A_2(0) = 0, A_1(0) \;\; = 0 \;\; \text{such that } g_0 \;\; A_1(0) = 1 \;\; L(0), A_2(0) = 0 \;\; = (1 - \mu_1(0))\mu_2(0)$$

- continuation of only long-acting insulin therapy in any given quarter t after the first quarter of follow-up among patients not right-censored in quarter t and who were previously continuously exposed to only long-acting insulin (PS denoted by $\mu_3(t)$):

$$g_0 \;\; A_1(t) = 0 \;\; \bar{L}(t), \bar{Y}(t) = 0, \bar{A}_2(t) = 0, \bar{A}_1(t-1) = 0$$

- switching to only long-acting insulin therapy in any given quarter t after the first quarter of follow-up among patients not right-censored in quarter t and who previously initiated short-acting insulin within the first 4 quarters of follow-up and who remained continuously exposed to both long-acting and short-acting insulin thereafter through quarter $t-1$ (PS denoted by $\mu_4(t)$):

$$g_0 \;\; A_1(t) = 0 \;\; \bar{L}(t), \bar{Y}(t) = 0, \bar{A}_2(t) = 0, \bar{A}(t-1) = \bar{a}^x(t-1), A_1(t-1) = 1 \;\; \text{with } x = 1$$

- continuation of both long-acting and short-acting insulin therapy in any given quarter t after the first quarter of follow-up among patients not right-censored in quarter t and who previously initiated short-acting insulin within the first 4 quarters of follow-up and who remained continuously exposed to both long-acting and short-acting insulin thereafter through quarter $t{-}1$ and who were not exposed to only long-acting insulin at quarter t (PS denoted by $\mu_5(t)$):

$$g_0 \;\; A_1(t) = 1 \;\; \bar{L}(t), \bar{Y}(t) = 0, \bar{A}_2(t) = 0, \bar{A}(t-1) = \bar{a}^x(t-1), A_1(t-1) = 1, A_1(t) \;\; = 0 \;\; \text{such that}$$

$$g_0 \;\; A_1(t) = 1 \;\; \bar{L}(t), \bar{Y}(t) = 0, \bar{A}_2(t) = 0, \bar{A}(t-1) = \bar{a}^x(t-1), A_1(t-1) = 1) = (1 - \mu_4(t))\mu_5(t)$$

- exposure to both long-acting and short-acting insulin in any given quarter t after the first quarter of follow-up among patients not right-censored in quarter t and who were previously continuously exposed to only long-acting insulin except at quarter t (PS denoted by $\mu_6(t)$):

$$g_0 \;\; A_1(t) = 1 \;\; \bar{L}(t), \bar{Y}(t) = 0, \bar{A}_2(t) = 0, \bar{A}_1(t-1) = 0, A_1(t) \;\; = 0 \;\; \text{such that}$$

$$g_0 \;\; A_1(t) = 1 \;\; \bar{L}(t), \bar{Y}(t) = 0, \bar{A}_2(t) = 0, \bar{A}_1(t-1) = 0 \;\; = (1 - \mu_3(t))\mu_6(t)$$

- right-censoring due to administrative end of study at any given quarter t (PS denoted by $\mu_7(t)$):

$$g_0 \;\; I \;\; A_2(t) = 1, \Gamma = 1 \;\; = 1 \;\; \bar{L}(t), \bar{Y}(t) = 0, \bar{A}_2(t-1) = 0, \bar{A}_1(t-1)$$

©2021 Shroeder EB et al. *JAMA Network Open*.

- right-censoring due to disenrollment from the health plan at any given quarter t among patients who were not right-censored at time t due to administrative end of study (PS denoted by $\mu_8(t)$):

$$g_0\left(I\left(A_2(t)=1,\Gamma=2\right)=1 \mid \bar{L}(t),\bar{Y}(t)=0,\bar{A}_2(t-1)=0,\bar{A}_1(t-1),I\left(A_2(t)=1,\Gamma=1\right)=0\right),$$

where $I\left(A_2(t)=1,\Gamma=j\right)$ is the indicator that the patient experienced the jth type of right-censoring event

- right-censoring due to start of pregnancy at any given quarter t among patients who were not right-censored at time t due to administrative end of study or health plan disenrollment (PS denoted by $\mu_9(t)$):

$$g_0\left(I\left(A_2(t)=1,\Gamma=3\right)=1 \mid \bar{L}(t),L_{♀}(0)=1,\bar{Y}(t)=0,\bar{A}_2(t-1)=0,\bar{A}_1(t-1),I\left(A_2(t)=1,\Gamma\in\{1,2\}\right)=0\right)$$

where $L_{♀}(0)$ denotes the indicator that the patient is female and $I\left(A_2(t)=1,\Gamma\in\{1,\ldots,k\}\right)=0$ is shorthand notation for $I\left(A_2(t)=1,\Gamma=1\right)=0,\ldots,I\left(A_2(t)=1,\Gamma=k\right)=0$

- right-censoring due to initiation of a non-standard insulins (animal or inhaled) at any given quarter t among patients who were not right-censored at time t due to administrative end of study, health plan disenrollment, or start of pregnancy (PS denoted by $\mu_{10}(t)$):

$$g_0\left(I\left(A_2(t)=1,\Gamma=4\right)=1 \mid \bar{L}(t),\bar{Y}(t)=0,\bar{A}_2(t-1)=0,\bar{A}_1(t-1),I\left(A_2(t)=1,\Gamma\in\{1,\ldots,3\}\right)=0\right)$$

- right-censoring due to death at any given quarter t among patients who were not right-censored at time t due to administrative end of study, health plan disenrollment, start of pregnancy, or initiation of non-standard insulins (PS denoted by $\mu_{11}(t)$):

$$g_0\left(I\left(A_2(t)=1,\Gamma=5\right)=1 \mid \bar{L}(t),\bar{Y}(t)=0,\bar{A}_2(t-1)=0,\bar{A}_1(t-1),I\left(A_2(t)=1,\Gamma\in\{1,\ldots,4\}\right)=0\right).$$

We note that the probability of a patient not experiencing a right-censoring event at quarter t given past covariates and exposures can then be derived from the last 5 PS as follows:

$$g_0\left(A_2(t)=0 \mid \bar{L}(t),\bar{Y}(t)=0,\bar{A}_2(t-1)=0,\bar{A}_1(t-1)\right)=\left(1-\mu_9(t)\right)^{L_{♀}(0)}\prod_{k\in\{7;8;10;11\}}\left(1-\mu_k(t)\right).$$

For the AMI, CHF, CVA, and CVD mortality outcomes, we constructed the denominators of the IP weights (1) for all outcomes contributing to the MSM fits (i.e., when $\prod_{j=0}^{t} g_x^*(A(j)\mid \bar{L}(j),\bar{A}(j-1))>0$) using the formulas below and estimates of the 11 PS above for $t=0,\ldots,15$:

- for replicates of person-time observations indexed by $x=0$:

$$\mu_1(0)\prod_{j=1}^{t}\mu_3(j)\left(1-\mu_9(j)\right)^{L_{♀}(0)}\prod_{k\in\{7;8;10;11\}}\left(1-\mu_k(j)\right),$$

where the product terms indexed by j are nil when $t=0$.

- for replicates of person-time observations indexed by $x=1$:

$$\mu_1(0)^{1-A_1(0)}\left(\left(1-\mu_1(0)\right)\mu_2(0)\right)^{A_1(0)}\prod_{j=1}^{t}\mu_3(j)^{(1-A_1(j))(1-A_1(j-1))}\mu_4(j)^{(1-A_1(j))A_1(j-1)}\times$$
$$\left(\left(1-\mu_3(j)\right)\mu_6(j)\right)^{A_1(j)(1-A_1(j-1))}\left(\left(1-\mu_4(j)\right)\mu_5(j)\right)^{A_1(j)A_1(j-1)}\left(1-\mu_9(j)\right)^{L_{♀}(0)}\prod_{k\in\{7;8;10;11\}}\left(1-\mu_k(j)\right),$$

where the product terms indexed by j are nil when $t=0$.

©2021 Shroeder EB et al. *JAMA Network Open*.

For the all-cause mortality outcome, the same formulas for the denominators of the IP weights were used except that the factors involving the PS for death (i.e., $(1-\mu_{11}(j))$) were ignored (i.e., replaced by 1's) because death is then the failure outcome of interest (i.e., there is no right-censoring due to death).

Each of the first three approaches considered for estimating the denominators of the IP weights using the formulas just described consists in fitting a separate logistic model for each of the the 11 PS $\mu_j(t)$ defined above. The three approaches only differ by the set of covariates that define each of the main terms included in each logistic model. We describe these sets in the next section.

eMethods 5 - Standard Propensity Score Estimation with Three Covariate Adjustment Sets

In the first approach implemented to estimate the denominators of the IP weights, the main terms included in a given PS logistic model were those associated with covariates presumed to impact both failure and the PS outcome as indicated in eTables 4-5. For instance, in the analyses of CHF, the PS logistic model for continuation of only long-acting insulin therapy in the first quarter of follow-up ($\mu_1(0)$) included main terms for all covariates in these tables where a value ≥ 1 is found in both the treatment and CHF columns. For the time-dependent covariates selected based on this rationale, only main terms for their current values $L(t)$ were included in the PS logistic models, i.e., no main terms for other summary measures of the covariate histories were considered (e.g., latest change in value $L(t) - L(t-1)$ or a lagged value $L(t-1)$). All PS logistic models fitted with pooled data over time (i.e., $\mu_j(t)$ for $j = 3, \dots, 11$) also included main terms for time t (expressed in 90-day intervals). PS logistic models for right-censoring events (i.e., $\mu_j(t)$ for $j = 7, \dots, 11$) included two main terms indicating whether the patient followed one of the two interventions (g_0^* or g_1^*) through quarter $t-1$. For the PS logistic models for administrative end of study ($\mu_7(t)$) and for the initiation of non-standard insulins ($\mu_{10}(t)$), only main terms for time t and the two indicators of prior exposures being consistent with interventions g_0^* and g_1^* were included in the model. For the PS logistic model for start of pregnancy ($\mu_9(t)$), only main terms for time t, age at index date, and the two indicators of prior exposures being consistent with interventions g_0^* and g_1^* were included in the model. All continuous variables considered by the various PS logistic models were discretized using the cutoffs given in eTable 6 and main terms for the resulting dummy variables (for the non-reference level) were included in the models. eTable 7 provides an example of the logistic model fit for $\mu_1(0)$ based on the PS estimation approach 1.

The second approach implemented to estimate the denominators of the IP weights followed the same principles with the difference that the main terms included in a given PS logistic model (including for start of pregnancy ($\mu_9(t)$) and administrative end of study ($\mu_7(t)$) were those associated with covariates presumed to, at least, impact failure as indicated in eTables 4-5. However, for the PS logistic model for the initiation of non-standard insulins ($\mu_{10}(t)$), only main terms for t and the two indicators of prior exposures being consistent with interventions g_0^* and g_1^* were included in the model because <5 patients initiated non-standard insulins which limited the number of covariate that could be considered. All other modeling decisions were identical to those of the first approach described above. eTables 8-9 provide an example of the logistic model fit for $\mu_1(0)$ based on the PS estimation approach 2.

The third approach implemented to estimate the denominators of the IP weights followed the same principles with the difference that the main terms included in a given PS logistic model were those associated with the covariates presumed to impact either failure or the PS outcome as indicated in eTables 4-5. The PS logistic models for treatment decisions (i.e., $\mu_j(t)$ for $j = 1, \dots, 6$) also included interaction terms between the study site indicators and index year indicators. The PS logistic models for the start of pregnancy ($\mu_9(t)$) and administrative end of study ($\mu_7(t)$) included main terms for all covariates presumed to affect failure. However, for the PS logistic model for the initiation of non-standard insulins ($\mu_{10}(t)$), only main terms for t and the two indicators of prior exposures being consistent with interventions g_0^* and g_1^* were included in the model because <5 patients initiated non-standard insulins which limited the number of covariate that could be considered. All other modeling decisions were identical to those of the first approach described above. eTables 10-12 provide an example of the logistic model fit for $\mu_1(0)$ based on the PS estimation approach 3.

Thus, the three sets of variables that define the main terms included in any given PS logistic model according to the three approaches just described are nested and of increasing size.

eMethods 6 - Data-adaptive Propensity Score Estimation

In the fourth approach implemented to estimate the denominators of the IP weights, a separate super learner[8] was used to estimate each of the 10 PS $\mu_j(t)$ with $j = 1, \dots, 9, 11$ instead of a separate logistic model (as done in the

©2021 Shroeder EB et al. *JAMA Network Open*.

first three approaches). Because <5 patients initiated non-standard insulins, the same logistic model for estimating $\mu_{10}(t)$ (initiation of non-standard insulins) as the one used in the prior three approaches was also used in approach 4. Each super learner was constructed based on 10-fold cross-validation and the following 15 learners:

- the same logistic model as in approach 1
- the same logistic model as in approach 2
- the same logistic model as in approach 3
- a logistic model constructed using the same principles described for approach 2 but with the difference that only main terms for covariates presumed to at least impact failure as indicated by a rank or 1 in eTables 4-5
- a logistic model constructed using the same principles described for approach 2 but with the difference that only main terms for covariates presumed to at least impact failure as indicated by a rank or 1 or 2 in eTables 4-5
- a logistic model constructed by including main terms for the first 30 covariates most associated with the PS outcome, i.e., the 30 covariates with the smallest p-values for the test that the Pearson's product moment correlation coefficient is equal to 0 (implemented by the screen.corRank screener in the SuperLearner R package[?])
- a logistic model constructed by including main terms for the first 20 covariates most associated with the PS outcome (selected by the screen.corRank screener)
- a logistic model constructed by including main terms for the first 10 covariates most associated with the PS outcome (selected by the screen.corRank screener)
- a logistic model constructed by including main terms for the first 5 covariates most associated with the PS outcome (selected by the screen.corRank screener)
- a regression based on linear splines and their tensor products that considers only the first 10 covariates most associated with the PS outcome (selected by the screen.corRank screener). The regression is implemented by the SL.polyclass routine given below that implements the polyclass learner[9] based on the Bayesian Information Criterion (BIC) as the model selection criterion. To improve computing speed, this learner was favored over the SL.polymars routine that is available by default in the SuperLearner R package but that relies on cross-validation for model selection.

```
SL.polyclass <- function (Y, X, newX, family, obsWeights, ...)
{
  tryCatch(require(polspline), warning = function(...) {
    stop("you have selected polyclass as a library algorithm but do not have
                                              the polspline package installed")
  })
  if (family$family == "gaussian") {
    stop("the outcome must be categorical")
  }
  if (family$family == "binomial") {
    fit.polyclass <- polyclass(Y, X, penalty = log(length(Y)), weight = obsWeights)
    out <- ppolyclass(cov = newX, fit = fit.polyclass)[, 2]
    fit <- list(fit = fit.polyclass)
  }
  foo <- list(pred = out, fit = fit)
  class(foo$fit) <- c("SL.polymars")
  return(foo)
}
```

- a regression based on linear splines and their tensor products that considers only the first 5 covariates most associated with the PS outcome (selected by the screen.corRank screener). The regression is implemented by the SL.polyclass routine given above that implements the polyclass learner[9] based on the Bayesian Information Criterion (BIC) as the model selection criterion.
- a random forest regression (implemented by the SL.randForest routine) that considers only the first 10 covariates most associated with the PS outcome (selected by the screen.corRank screener)
- a random forest regression (implemented by the SL.randForest routine) that considers only the first 5 covariates most associated with the PS outcome (selected by the screen.corRank screener)
- an extreme gradient boosting regression (implemented by the SL.xgboost routine) that considers only the first 10 covariates most associated with the PS outcome (selected by the screen.corRank screener)
- an extreme gradient boosting regression (implemented by the SL.xgboost routine) that considers only the first 5 covariates most associated with the PS outcome (selected by the screen.corRank screener)

eTable 13 provides an example of the super learner fit for $\mu_1(0)$ based on the PS estimation approach 4.

eMethods 7 - Results

eTable 14 describes the distribution of reasons for end of follow-up in all primary analyses.

The counts of patients following each exposure regimen at each quarter of follow-up in the AMI and CVD mortality analyses are described by the histograms in eFigure 1. These counts were similar for the CHF, CVA, and all-cause mortality analyses (data not shown). We note that it is possible for the same patient to follow both exposure regimens simultaneously during the first 3 quarters of follow-up only.

Results of analyses implemented with the four PS estimation approaches described above along with their corresponding unadjusted analyses (i.e., same models fitted without weights) are displayed in eTables 15, 16, 17, 18, and 19 for AMI, CHF, CVA, CVD-mortality, and all-cause mortality, respectively. Inference for the hazard ratio is given in the column “HR” and derived from the MSM fit that assumes constant hazard ratios over time (proportionality assumption). Inference in the “AUC”, “RD1”, “RD2”, “RD3”, and “RD4” columns are derived from the same saturated MSM fit. The “AUC” column contains the p-value from the statistical test that the area between the survival curves is equal to 0. The “RD1”, “RD2”,“RD3”, and “RD4” columns provide inferences for the cumulative risk differences at 1, 2, 3, and 4 years (i.e., 4, 8, 12, and 16 quarters) after the index date, respectively. 95% confidence intervals for the HR and RDs are given in between squared brackets, standard errors are given by “SE”, and the p-values of the statistical tests that HR=1/RD=0 are given by “p”. The cells highlighted in yellow indicate p<0.05, i.e., statistically significant finding. We note that p-values were not adjusted for multiple testing. The crude (i.e., unadjusted) and SL-based IPW estimates of the counterfactual survival curves associated with the AUC p-values given in eTables 15-19 are displayed in eFigures 2-3. Summary statistics for the inverse probability weights involved in all analyses are displayed in eTable 20.

©2021 Shroeder EB et al. *JAMA Network Open*.

eTable 1: Sources of Data and Codes Used to Ascertain Major Cardiovascular Events and Mortality.

**Fatal or Nonfatal Myocardial Infarction** (including Acute Coronary Syndrome)	ICD-9-CM codes : 410.xx	Inpatient hospital discharges (principle discharge diagnosis)
**Fatal or Nonfatal Stroke** Ischemic stroke Hemorrhagic stroke	ICD-9-CM codes : 430.xx, 431.xx, 433.x1, 434.x1	Inpatient hospital discharges (principle discharge diagnosis)
**Hospitalization for Heart Failure** **(**discharged either alive or deceased)	ICD-9-CM codes: 402.01, 402.11, 402.91, 404.01, 404.03, 404.11, 404.13, 404.91, 404.93, 428.xx	Inpatient hospital discharges (principle discharge diagnosis)
**Cardiovascular Mortality**	ICD9/ICD10 groups:<br>CHD,HF: 50,51,52,53,54,55,58,59,60<br>CeVD: 61<br>PAD/Arteriosclerosis: 62,63	Social Security Admin;<br>National Death Index;<br>State Death Records;<br>Tumor Registry data;<br>Encounter data;<br>Patient data;<br>Membership data
**Overall Mortality**	--	Social Security Admin;<br>National Death Index;<br>State Death Records;<br>Tumor Registry data;<br>Encounter data;<br>Patient data;<br>Membership data
CHD: coronary heart disease; HF: heart failure; CeVD: cerebrovascular disease; PAD: peripheral arterial disease.		

©2021 Shroeder EB et al. *JAMA Network Open*.

eTable 2: Part I of II - Brief description of all attributes (L) in the covariate adjustment sets.

Covariate handle	Brief covariate definition
afib	atrial fibrillation
age.at.a1c	age at first elevated a1c (i.e., index date)
alcoholabuse	alcohol abuse
anemia	anemia
anticoag	anticoagulant medication
anxiety	anxiety
asthma	asthma
bariatric	bariatric surgery
bipolar	bipolar affective disorder
bmi	body mass index (Kg/m2)
cabg	coronary artery bypass graft
cad	coronary artery disease
cancer	cancer other than non-melanoma skin cancer
census.hsgrad	high school graduate
census.medhhincome	median household income
cevd	cerebrovascular disease
chf	congestive heart failure
chf.event	CHF hospitlaization
ckd	chronic kidney disease
composite.protein	urine microalbumin creatinine ratio
connective	vasculitis/connective tissue disease
copd	chronic obstructive lung disease
dementia	dementia
depression	depression
diastolic	diastolic blood pressure
dpp4	DPP-4 class of glucose-lowering medication
drugabuse.alt	substance abuse disorder (other than alcohol)
drugcount	total number of prescription medications
early.adopter	use of DPP-4, GLT-1, SGLTs within 5 years from FDA approval
elixhauser	Elixhauser comorbidity score
endovisit	indicator of outpatient visit with an endocrinologist
flag.incident	incident diabetes (diabetes recognition date $\geq$ 18 months since health plan enrollment)
gender	gender
gfr	glomerular filtration rate cc/min/2.72m2
glp1	GLP-1 agonist glucose lowering medication
hdl	high-denisty lipoprotein cholesterol

eTable 3: Part II of II - Brief description of all attributes (L) in the covariate adjustment sets.

Covariate handle	Brief covariate definition
hgba1c	hemoglobin A1c value (%)
hiv	HIV infection
hmosite	study site
htnmed	hypertension medication
hyperglycemia	hyperglycemia diagnosis code
hyperlipidemia	dyslipidemia
hypertension	hypertension
hypoglycemia	hypoglycemia diagnosis code
index.year	index year
insulin.rxmd.spec	prescribing provider specialty for insulin dispensed at index
insulin.rxmd.type	index insulin provider type (NP/PA versus MD/PO)
insulin.rxmd.yrs	years since prescribing provider of index insulin graduated
ip.count	number of inpatient encounters
ldl	low-denisty lipoprotein cholesterol
lipidmed	cholesterol medication
mavalvedisorder	mitral or aortic valve heart disease
met	metformin glucose lowering medication
mi.event	myocardial infarction
neurodisorder	neuromuscular disorder
nitrate	nitrate medication
platemed	platelet inhibitor medication
ptvalvedisorder	pulmonic or tricuspid valve heart disease
pvd	peripheral vascular disease
racegrp	race group
retinopathy	retinopathy
schizophrenia	schizophrenia
sglt2	SGLT2 inhibitor class of glucose lowering medication
smoking.status	smoking status
stent	stent placed in coronary artery
stroke.event	stroke event
sul	sulfonylurea glucose lowering medication
systolic	systolic blood pressure
tzd	TZD glucose lowering medication
years.since.dm	duration of diabetes in years

©2021 Shroeder EB et al. *JAMA Network Open*.

eTable 4: Part I of II - List of covariates considered in the various analyses and whether they are assumed to impact treatment decisions, censoring events, or outcomes. A value of 0 encodes the assumption of no covariate impact. A covariate with rank 1 is assumed to be more impactful than a covariate with rank 2 which is assumed to be more impactful than a covariate with rank 3.

Covariate	AMI	CHF	CVA	CVD	Death $\mu_{11}(0)$	Treatment $\mu_j(t)$ for $j = 1,...,6$	Insurance coverage $\mu_8(t)$	Time-dependent
afib	3	2	1	3	3	0	0	1
age.at.a1c	1	1	1	1	1	1	1	0
alcoholabuse	2	2	2	2	2	1	1	1
anemia	3	2	3	3	3	0	0	1
anticoag	3	2	1	1	1	0	0	1
anxiety	3	3	3	3	3	1	0	1
asthma	3	3	3	3	3	0	0	1
bariatric	1	0	3	0	0	0	1	1
bipolar	2	3	3	1	1	1	1	1
bmi	3	3	3	3	3	0	0	1
cabg	1	1	1	1	1	1	1	1
cad	1	1	1	1	1	1	1	1
cancer	0	0	0	0	0	0	1	1
census.hsgrad	3	3	3	3	3	1	1	0
census.medhhincome	3	3	3	3	3	1	1	0
cevd	1	1	1	1	1	1	1	1
chf	1	1	1	1	1	1	1	1
chf.event	1	1	1	1	1	1	1	1
ckd	3	3	3	3	3	0	1	1
composite.protein	3	3	3	3	3	0	0	1
connective	2	2	2	2	2	0	1	1
copd	3	2	3	2	2	0	1	1
dementia	3	3	2	3	3	1	1	1
depression	2	2	2	2	2	1	1	1
diastolic	2	1	1	0	0	0	0	1
dpp4	3	3	3	3	3	1	0	1
drugabuse.alt	2	3	2	1	1	1	1	1
drugcount	0	0	0	0	0	1	0	0
early.adopter	0	0	0	0	0	1	0	0
elixhauser	0	0	0	0	0	1	1	0
endovisit	0	0	0	0	0	1	0	1
flag.incident	2	2	2	2	2	0	0	0
gender	1	1	1	1	1	1	0	0
gfr	1	1	1	1	1	0	0	1
glp1	3	3	3	3	3	1	0	1
hdl	1	0	1	1	0	0	0	1

©2021 Shroeder EB et al. *JAMA Network Open*.

eTable 5: Part II of II - List of covariates considered in the various analyses and whether they are assumed to impact treatment decisions, censoring events, or outcomes. A value of 0 encodes the assumption of no covariate impact. A covariate with rank 1 is assumed to be more impactful than a covariate with rank 2 which is assumed to be more impactful than a covariate with rank 3.

Covariate	AMI	CHF	CVA	CVD	Death $\mu_{11}(0)$	Treatment $\mu_j(t)$ for $j = 1, \ldots, 6$	Insurance coverage $\mu_8(t)$	Time-dependent
hgba1c	1	1	1	1	1	1	0	1
hiv	2	2	2	2	2	0	1	1
hmosite	0	0	0	0	0	1	1	0
htnmed	1	1	1	3	0	0	0	1
hyperglycemia	3	3	3	3	3	1	0	1
hyperlipidemia	1	1	1	1	1	0	0	1
hypertension	1	1	1	1	1	0	1	1
hypoglycemia	3	3	3	3	3	1	0	1
index.year	0	0	0	0	0	1	0	0
insulin.rxmd.spec	0	0	0	0	0	1	0	0
insulin.rxmd.type	0	0	0	0	0	1	0	0
insulin.rxmd.yrs	0	0	0	0	0	1	0	0
ip.count	1	1	1	1	1	0	1	1
ldl	1	0	1	1	0	0	0	1
lipidmed	1	0	1	1	0	0	0	1
mavalvedisorder	0	1	1	2	2	0	0	1
met	3	3	3	3	3	1	0	1
mi.event	1	1	1	1	1	0	1	0
neurodisorder	0	0	0	0	0	0	1	1
nitrate	1	1	2	1	1	0	0	1
platemed	1	2	1	1	1	0	0	1
ptvalvedisorder	0	1	0	2	2	0	0	1
pvd	1	1	1	1	1	0	1	1
racegrp	1	1	1	1	1	1	1	0
retinopathy	2	2	2	2	2	0	0	1
schizophrenia	2	3	3	1	1	1	1	1
sglt2	3	3	3	3	3	1	0	1
smoking.status	1	1	1	1	1	1	0	0
stent	1	1	1	1	1	1	1	1
stroke.event	1	1	1	1	1	1	1	1
sul	3	3	3	3	3	1	0	1
systolic	1	1	1	2	0	0	0	1
tzd	3	3	3	3	3	1	0	1
years.since.dm	2	2	2	2	2	0	0	0

©2021 Shroeder EB et al. *JAMA Network Open*.

eTable 6: Cutoffs used to discretize continuous covariates.

Variable	Cutoffs
age.at.a1c (years)	35;45;55;65;75
bmi (Kg/m^2)	18.5; 25; 30; 35; 40
census.hsgrad	0.5
census.medhhincome	30000;50000;70000;90000
diastolic (mm Hg)	80;90;100
drugcount	2;3;4;5;6;7;8;9
elixhauser score	1;3;5
gfr (mL/1.73 m^2/min)	15; 30; 45; 60; 90
hdl (mg/dL)	40;50;60
hgba1c (%)	7; 7.5; 8; 8.5;9;10
insulin.rxmd.yrs	5;20
ip.count	1;2
ldl (mg/dL)	70;100;130
systolic (mm Hg)	120;140;160
t	1;2;3;4;6;8;10;12;16
years.since.dm	1;6;10

©2021 Shroeder EB et al. *JAMA Network Open*.

eTable 7: PS estimation approach 1 in the AMI analysis: Logistic model for the probability of continuing exposure to long-acting only insulin during first follow-up period given baseline covariates. Model fitted with 54269 observations from 54269 unique patients. Reference categories: age.at.a1c.geq.55.sl.65, hgba1c.geq.7.sl.7.5, genderM, racegrp6:WHITE, census.medhhincome.geq.50000.sl.70000, census.hsgrad.geq.0.5, smoking.statusNEVER/UNK. Indicators of missing covariate measurement are denoted by I.* (e.g., I.census.hsgrad monitoring denotes the absence of a census.hsgrad measurement at quarter ’0’). The term for sglt2 use is omitted from the output below because no patient used this drug at study entry.

Covariate	Coef	OR
(Intercept)	2.585	
age.at.a1c <35	-0.455	0.635
age.at.a1c in [35;45[	-0.039	0.962
age.at.a1c in [45;55[	0.021	1.022
age.at.a1c in [65;75[	-0.018	0.982
age.at.a1c ≥75	-8e-02	0.923
dementia	-0.27	0.763
dpp4	-0.247	0.781
glp1	-0.613	0.542
met	-0.061	0.941
sul	-0.179	0.836
tzd	-0.181	0.835
hgba1c <7	-0.112	0.894
hgba1c in [7.5;8[	0.016	1.016
hgba1c in [8;8.5[	-0.087	0.917
hgba1c in [8.5;9[	-0.276	0.759
hgba1c in [9;10[	-15.658	0
hgba1c ≥10	-15.475	0
hyperglycemia	-0.358	0.699
hypoglycemia	-0.51	0.6
genderF	-5e-03	0.995
racegrp1:HISPANIC	-0.192	0.826
racegrp2:BLACK	-0.276	0.759
racegrp3:HI/PI	-0.243	0.784
racegrp4:ASIAN	-0.356	0.7

Covariate	Coef	OR
racegrp5:NATIV	-0.148	0.862
racegrp7:MISS	0.054	1.055
census.medhhincome <30000	-0.129	0.879
census.medhhincome in [30000;50000[	-0.036	0.965
census.medhhincome in [70000;90000[	-0.021	0.979
census.medhhincome ≥90000	0.011	1.011
census.hsgrad <0.5	-0.092	0.912
smoking.statusCURRENT	-0.019	0.981
smoking.statusPAST	0.042	1.043
alcoholabuse	-0.113	0.893
anxiety	-0.126	0.881
bipolar	-0.216	0.806
cabg	-0.502	0.605
cad	-0.045	0.956
cevd	-0.218	0.804
chf	-0.125	0.883
depression	-0.197	0.821
drugabuse.alt	-0.153	0.858
schizophrenia	-0.128	0.88
stent	0.131	1.14
chf.event	-0.507	0.602
stroke.event	-0.233	0.792
I.census.hsgrad	-0.428	0.652
I.census.medhhincome	0.259	1.295

©2021 Shroeder EB et al. *JAMA Network Open*.

eTable 8: PS estimation approach 2 in AMI analysis (Part I of II): Logistic model for the probability of continuing exposure to long-acting only insulin during first follow-up period given baseline covariates. Model fitted with 54269 observations from 54269 unique patients. Reference categories: age.at.a1c.geq.55.sl.65, genderM, racegrp6:WHITE, smoking.statusNEVER/UNK, ip.count.sl.1, gfr.geq.60.sl.90, hdl.sl.40, hgba1c.geq.7.sl.7.5, ldl.geq.70.sl.100, systolic.geq.120.sl.140, years.since.dm.geq.6.sl.10, flag.incidentUnknown, diastolic.sl.80, census.medhhincome.geq.50000.sl.70000, census.hsgrad.geq.0.5, bmi.geq.30.sl.35, composite.protein1:NORML. Indicators of missing covariate measurement are denoted by I.* (e.g., I.census.hsgrad monitoring denotes the absence of a census.hsgrad measurement at quarter ’0’). The term for sglt2 use is omitted from the output below because no patient used this drug at study entry.

Covariate	Coef	OR
(Intercept)	2.621	
age.at.a1c <35	-0.418	0.659
age.at.a1c in [35;45[	-0.036	0.965
age.at.a1c in [45;55[	3e-02	1.031
age.at.a1c in [65;75[	0.016	1.016
age.at.a1c ≥75	0.019	1.02
genderF	0.034	1.034
racegrp1:HISPANIC	-0.17	0.844
racegrp2:BLACK	-0.228	0.797
racegrp3:HI/PI	-0.185	0.831
racegrp4:ASIAN	-0.26	0.771
racegrp5:NATIV	-0.124	0.883
racegrp7:MISS	0.073	1.075
smoking.statusCURRENT	-0.015	0.986
smoking.statusPAST	0.029	1.029
mi.event	0.236	1.266
bariatric	-15.456	0
cabg	-0.343	0.71
cad	0.027	1.027
cevd	-0.025	0.975
chf	-0.12	0.887
hyperlipidemia	0.216	1.242
hypertension	-5e-03	0.995
pvd	-0.047	0.955
stent	0.288	1.334
chf.event	-0.12	0.886
htnmed	0.071	1.074

Covariate	Coef	OR
ip.count in [1;2[	-0.314	0.73
ip.count ≥2	-0.687	0.503
lipidmed	-0.111	0.895
nitrate	8e-03	1.008
platemed	-0.096	0.909
gfr <15	-1.101	0.333
gfr in [15;30[	-0.262	0.77
gfr in [30;45[	-4e-02	0.96
gfr in [45;60[	-0.021	0.979
gfr ≥90	-0.037	0.964
hdl in [40;50[	-0.018	0.982
hdl in [50;60[	-0.049	0.953
hdl ≥60	-0.069	0.933
hgba1c <7	-0.093	0.911
hgba1c in [7.5;8[	0.011	1.011
hgba1c in [8;8.5[	-0.098	0.906
hgba1c in [8.5;9[	-0.291	0.748
hgba1c in [9;10[	-15.655	0
hgba1c ≥10	-15.301	0
ldl <70	0.023	1.024
ldl in [100;130[	-0.183	0.832
ldl ≥130	-0.352	0.703
stroke.event	-8e-02	0.923
systolic <120	-2e-03	0.998
systolic in [140;160[	-0.077	0.926
systolic ≥160	0.041	1.042

©2021 Shroeder EB et al. *JAMA Network Open*.

eTable 9: PS estimation approach 2 in AMI analysis (Part II of II): Logistic model for the probability of continuing exposure to long-acting only insulin during first follow-up period given baseline covariates. Model fitted with 54269 observations from 54269 unique patients. Reference categories: age.at.a1c.geq.55.sl.65, genderM, racegrp6:WHITE, smoking.statusNEVER/UNK, ip.count.sl.1, gfr.geq.60.sl.90, hdl.sl.40, hgba1c.geq.7.sl.7.5, ldl.geq.70.sl.100, systolic.geq.120.sl.140, years.since.dm.geq.6.sl.10, flag.incidentUnknown, diastolic.sl.80, census.medhhincome.geq.50000.sl.70000, census.hsgrad.geq.0.5, bmi.geq.30.sl.35, composite.protein1:NORML. Indicators of missing covariate measurement are denoted by I.* (e.g., I.census.hsgrad monitoring denotes the absence of a census.hsgrad measurement at quarter '0'). The term for sglt2 use is omitted from the output below because no patient used this drug at study entry.

Covariate	Coef	OR
I.gfr	-0.115	0.891
I.hdl	0.131	1.14
I.ldl	0.066	1.069
I.systolic	11.55	103744.696
years.since.dm <1	-0.139	0.87
years.since.dm in [1;6[	-0.21	0.811
years.since.dm ≥10	0.148	1.16
flag.incidentNo	0.172	1.187
flag.incidentYes	0.151	1.163
alcoholabuse	-3e-03	0.997
bipolar	-0.185	0.831
connective	-0.237	0.789
depression	-0.144	0.866
drugabuse.alt	-0.032	0.968
hiv	6e-03	1.006
retinopathy	-0.086	0.918
schizophrenia	-0.028	0.973
diastolic in [80;90[	0.017	1.017
diastolic in [90;100[	-0.084	0.919
diastolic ≥100	-0.223	0.8
I.diastolic	-11.449	0
census.medhhincome <30000	-0.111	0.895
census.medhhincome in [30000;50000[	-0.031	0.97
census.medhhincome in [70000;90000[	-0.027	0.974
census.medhhincome ≥90000	0.015	1.015
census.hsgrad <0.5	-0.082	0.922

Covariate	Coef	OR
afib	0.219	1.245
anxiety	-8e-02	0.923
asthma	-0.182	0.834
ckd	-0.118	0.888
copd	0.051	1.053
dementia	-0.039	0.962
anemia	-0.022	0.978
dpp4	-0.305	0.737
glp1	-0.642	0.526
met	-0.145	0.865
anticoag	-0.147	0.863
sul	-0.218	0.804
tzd	-0.157	0.855
bmi <18.5	-0.812	0.444
bmi in [18.5;25[	-0.325	0.722
bmi in [25;30[	-0.132	0.876
bmi in [35;40[	0.059	1.061
bmi ≥40	0.101	1.106
composite.protein0:UNK	0.014	1.014
composite.protein2:MICRO	0.127	1.135
composite.protein3:MACRO	0.16	1.174
hyperglycemia	-0.027	0.973
hypoglycemia	-0.353	0.702
I.census.medhhincome	0.362	1.436
I.census.hsgrad	-0.499	0.607
I.bmi	-0.19	0.827

©2021 Shroeder EB et al. *JAMA Network Open*.

eTable 10: PS estimation approach 3 in AMI analysis (Part I of III): Logistic model for the probability of continuing exposure to long-acting only insulin during first follow-up period given baseline covariates. Model fitted with 54269 observations from 54269 unique patients. Reference categories: age.at.a1c.geq.55.sl.65, insulin.rxmd.yrs.geq.5.sl.20, insulin.rxmd.specOTHER, insulin.rxmd.typeMD/DO, hgba1c.geq.7.sl.7.5, genderM, racegrp6:WHITE, census.medhhincome.geq.50000.sl.70000, census.hsgrad.geq.0.5, smoking.statusNEVER/UNK, elixhauser.geq.5, drugcount.geq.5.sl.6, ip.count.sl.1, gfr.geq.60.sl.90, hdl.sl.40, ldl.geq.70.sl.100, systolic.geq.120.sl.140, years.since.dm.geq.6.sl.10, flag.incidentUnknown, diastolic.sl.80, bmi.geq.30.sl.35, composite.protein1:NORML, hmositeKPSC. The term for sglt2 use is omitted from the output below because no patient used this drug at study entry.

Covariate	Coef	OR
(Intercept)	3.437	0
age.at.a1c <35	-0.293	0.746
age.at.a1c in [35;45[	0.037	1.037
age.at.a1c in [45;55[	0.064	1.066
age.at.a1c in [65;75[	-0.016	0.984
age.at.a1c ≥75	-0.054	0.947
early.adopter	0.161	1.175
insulin.rxmd.yrs <5	-0.021	0.979
insulin.rxmd.yrs ≥20	0.013	1.013
insulin.rxmd.specENDO	-2e-03	0.998
insulin.rxmd.specUNK	0.433	1.542
insulin.rxmd.typeNP/PA	0.012	1.012
insulin.rxmd.typeOTHER	-5e-03	0.995
insulin.rxmd.typeUNK	-0.357	0.7
dementia	-0.047	0.954
dpp4	-0.7	0.496
glp1	-0.977	0.377
met	-0.471	0.624
sul	-0.59	0.555
tzd	-0.34	0.712
hgba1c <7	-0.091	0.913
hgba1c in [7.5;8[	0.015	1.015
hgba1c in [8;8.5[	-0.103	0.902
hgba1c in [8.5;9[	-0.278	0.757
hgba1c in [9;10[	-15.739	0
hgba1c ≥10	-15.185	0
hyperglycemia	-0.027	0.973
hypoglycemia	-0.374	0.688
endovisit	-0.328	0.72
l.insulin.rxmd.yrs	0.155	1.168
genderF	-2e-03	0.998
racegrp1:HISPANIC	-0.118	0.889
racegrp2:BLACK	-0.239	0.788
racegrp3:HI/PI	-0.112	0.894
racegrp4:ASIAN	-0.195	0.823

Covariate	Coef	OR
racegrp5:NATIV	-0.132	0.876
racegrp7:MISS	0.139	1.15
census.medhhincome <30000	-0.122	0.885
census.medhhincome in [30000;50000[	-0.038	0.962
census.medhhincome in [70000;90000[	-0.017	0.983
census.medhhincome ≥90000	0.023	1.023
census.hsgrad <0.5	-0.057	0.945
smoking.statusCURRENT	0	1
smoking.statusPAST	1e-02	1.01
elixhauser in [1;3[	5e-02	1.051
elixhauser in [3;5[	0.011	1.011
drugcount <2	-1.934	0.144
drugcount in [2;3[	-1.245	0.288
drugcount in [3;4[	-0.764	0.466
drugcount in [4;5[	-0.403	0.669
drugcount in [6;7[	0.093	1.098
drugcount in [7;8[	0.332	1.394
drugcount in [8;9[	0.396	1.487
drugcount ≥9	0.379	1.461
alcoholabuse	-0.067	0.936
anxiety	-0.145	0.865
bipolar	-0.353	0.703
cabg	-0.308	0.735
cad	-0.022	0.978
cevd	-0.036	0.964
chf	-0.196	0.822
depression	-0.228	0.796
drugabuse.alt	-0.104	0.902
schizophrenia	-0.123	0.884
stent	0.302	1.353
chf.event	-0.146	0.864
stroke.event	-0.092	0.913
l.census.hsgrad	-0.642	0.526
l.census.medhhincome	0.415	1.514
mi.event	0.234	1.263

©2021 Shroeder EB et al. *JAMA Network Open*.

eTable 11: PS estimation approach 3 in AMI analysis (Part II of III): Logistic model for the probability of continuing exposure to long-acting only insulin during first follow-up period given baseline covariates. Model fitted with 54269 observations from 54269 unique patients. Reference categories: age.at.a1c.geq.55.sl.65, insulin.rxmd.yrs.geq.5.sl.20, insulin.rxmd.specOTHER, insulin.rxmd.typeMD/DO, hgba1c.geq.7.sl.7.5, genderM, racegrp6:WHITE, census.medhhincome.geq.50000.sl.70000, census.hsgrad.geq.0.5, smoking.statusNEVER/UNK, elixhauser.geq.5, drugcount.geq.5.sl.6, ip.count.sl.1, gfr.geq.60.sl.90, hdl.sl.40, ldl.geq.70.sl.100, systolic.geq.120.sl.140, years.since.dm.geq.6.sl.10, flag.incidentUnknown, diastolic.sl.80, bmi.geq.30.sl.35, composite.protein1:NORML, hmositeKPSC. The term for sglt2 use is omitted from the output below because no patient used this drug at study entry.

Covariate	Coef	OR
bariatric	-15.407	0
hyperlipidemia	0.14	1.15
hypertension	-0.041	0.96
pvd	-0.045	0.956
htnmed	-0.349	0.705
ip.count in [1;2[	-0.348	0.706
ip.count ≥2	-0.742	0.476
lipidmed	-0.338	0.713
nitrate	-0.086	0.918
platemed	-0.238	0.788
gfr <15	-1.263	0.283
gfr in [15;30[	-0.5	0.606
gfr in [30;45[	-0.179	0.836
gfr in [45;60[	-9e-02	0.914
gfr ≥90	0.014	1.014
hdl in [40;50[	1e-03	1.001
hdl in [50;60[	-0.021	0.979
hdl ≥60	-0.048	0.954
ldl <70	-0.013	0.987
ldl in [100;130[	-0.13	0.878
ldl ≥130	-0.227	0.797
systolic <120	-3e-03	0.997
systolic in [140;160[	-0.098	0.907
systolic ≥160	0.054	1.055
I.gfr	-0.033	0.967
I.hdl	0.112	1.119
I.ldl	0.015	1.015
I.systolic	12.528	275945.209
years.since.dm <1	-0.197	0.821
years.since.dm in [1;6[	-0.169	0.845
years.since.dm ≥10	0.092	1.097
flag.incidentNo	0.136	1.145
flag.incidentYes	0.084	1.088
connective	-0.391	0.677
hiv	-0.076	0.927

Covariate	Coef	OR
retinopathy	-0.069	0.933
diastolic in [80;90[	0.043	1.044
diastolic in [90;100[	-0.057	0.944
diastolic ≥100	-0.139	0.871
I.diastolic	-12.205	0
afib	0.187	1.206
asthma	-0.264	0.768
ckd	-0.145	0.865
copd	1e-03	1.001
anemia	-0.071	0.931
anticoag	-0.278	0.757
bmi <18.5	-0.759	0.468
bmi in [18.5;25[	-0.271	0.763
bmi in [25;30[	-0.093	0.912
bmi in [35;40[	0.021	1.021
bmi ≥40	0.041	1.042
composite.protein0:UNK	-0.023	0.977
composite.protein2:MICRO	0.112	1.118
composite.protein3:MACRO	0.142	1.153
I.bmi	-0.031	0.969
hmositeHPI	0.487	1.628
hmositeKPCO	0.275	1.317
hmositeKPNC	0.523	1.688
index.year2006	0.081	1.084
index.year2007	0.413	1.511
index.year2008	0.696	2.007
index.year2009	0.709	2.033
index.year2010	0.728	2.07
index.year2011	0.759	2.136
index.year2012	0.822	2.275
index.year2013	0.75	2.116
hmositeHPI:index.year2006	0.772	2.164
hmositeKPCO:index.year2006	0.26	1.297
hmositeKPNC:index.year2006	-0.088	0.916
hmositeHPI:index.year2007	-0.645	0.525

©2021 Shroeder EB et al. *JAMA Network Open*.

eTable 12: PS estimation approach 3 in AMI analysis (Part III of III): Logistic model for the probability of continuing exposure to long-acting only insulin during first follow-up period given baseline covariates. Model fitted with 54269 observations from 54269 unique patients. Reference categories: age.at.a1c.geq.55.sl.65, insulin.rxmd.yrs.geq.5.sl.20, insulin.rxmd.specOTHER, insulin.rxmd.typeMD/DO, hgba1c.geq.7.sl.7.5, genderM, racegrp6:WHITE, census.medhhincome.geq.50000.sl.70000, census.hsgrad.geq.0.5, smoking.statusNEVER/UNK, elixhauser.geq.5, drugcount.geq.5.sl.6, ip.count.sl.1, gfr.geq.60.sl.90, hdl.sl.40, ldl.geq.70.sl.100, systolic.geq.120.sl.140, years.since.dm.geq.6.sl.10, flag.incidentUnknown, diastolic.sl.80, bmi.geq.30.sl.35, composite.protein1:NORML, hmositeKPSC. The term for sglt2 use is omitted from the output below because no patient used this drug at study entry.

Covariate	Coef	OR
hmositeKPCO:index.year2007	0.012	1.012
hmositeKPNC:index.year2007	-0.428	0.652
hmositeHPI:index.year2008	-0.459	0.632
hmositeKPCO:index.year2008	-0.269	0.764
hmositeKPNC:index.year2008	-0.665	0.514
hmositeHPI:index.year2009	0.399	1.491
hmositeKPCO:index.year2009	0.013	1.013
hmositeKPNC:index.year2009	-0.541	0.582
hmositeHPI:index.year2010	-0.706	0.494
hmositeKPCO:index.year2010	-0.384	0.681
hmositeKPNC:index.year2010	-0.732	0.481
hmositeHPI:index.year2011	-0.17	0.843
hmositeKPCO:index.year2011	-0.019	0.982
hmositeKPNC:index.year2011	-0.727	0.484
hmositeHPI:index.year2012	-0.133	0.875
hmositeKPCO:index.year2012	-0.227	0.797
hmositeKPNC:index.year2012	-0.744	0.475
hmositeHPI:index.year2013	0.484	1.622
hmositeKPCO:index.year2013	-0.119	0.888
hmositeKPNC:index.year2013	-0.765	0.465

©2021 Shroeder EB et al. *JAMA Network Open*.

eTable 13: PS estimation approach 4 in AMI analysis: Super learner estimator for the probability of continuing exposure to long-acting only insulin during first follow-up period given baseline covariates. Estimators derived based on 54269 observations from 54269 unique patients. The weighted average (SL weights) of the 15 learners that define the super learner was constructed based on 10-fold cross-validation (CV).

	Logistic model 1 (eTable 7)	Logistic model 2 (eTables 8-9)	Logistic model 3 (eTables 10-11)
CV risk	0.09852	0.09762	0.09478
SL weights	0	0	0.89058

	Logistic model 4 Approach 2, rank=1 only	Logistic model 5 Approach 2, rank=1 or 2	Logistic model 6 30 covariates, correlation ranks
CV risk	0.09806	0.09795	0.09614
SL weights	0	0	0.04315

	Logistic model 7 20 covariates, correlation ranks	Logistic model 8 10 covariates, correlation ranks	Logistic model 9 5 covariates, correlation ranks
CV risk	0.09644	0.09735	0.09781
SL weights	0	0	0.00123

	Polyclass 1 10 covariates, correlation ranks	Polyclass 2 5 covariates, correlation ranks	Random Forest 1 10 covariates, correlation ranks
CV risk	0.09774	0.09801	0.11107
SL weights	0.03643	0	0.00706

	Random Forest 2 5 covariates, correlation ranks	xgboost 1 10 covariates, correlation ranks	xgboost 2 5 covariates, correlation ranks
CV risk	0.1113	0.09828	0.09852
SL weights	0.02155	0	0

©2021 Shroeder EB et al. *JAMA Network Open*.

eTable 14: Event rates and reasons for end of analytic follow-up.

	Study Events				
	MI (n=57,278)	Mortality (n=57,278)	CVA (n=57,278)	CHF (n=57,278)	CVD Mortality (n=39,279)
Administrative end of follow-up, No. (%)	44,299 (77.3)	45,235 (79.0)	44,605 (77.9)	44,131 (77.0)	32,497 (82.7)
End enrollment in health or pharmacy insurance, No. (%)	8,207 (14.3)	8,253 (14.4)	8,242 (14.4)	8,176 (14.3)	4,735 (12.1)
Start of pregnancy, No. (%)	-- (0.3)	-- (0.3)	-- (0.3)	-- (0.3)	-- (0.2)
Death as a right-censoring event, No. (%)	3,139 (5.5)	NA	3,247 (5.7)	2,755 (4.8)	1,103 (2.8)
Switch to inhaled/animal insulin, No. (%)	-- (0.0)	-- (0.0)	-- (0.0)	-- (0.0)	-- (0.0)
Outcome, No. (%)	1,457 (2.5)	3,612 (6.3)	1,006 (1.8)	2,040 (3.6)	843 (2.1)
Event rates by exposure, LA+SA group[a]/LA group[a]					
No. with outcome	323/618	675/1152	215/422	541/919	200/308
Person-time in quarters	158,799/324,000	160,205/327,686	159,152/325,199	158,394/323,913	104,425/193,432

Numbers less than 200 are masked to prevent reporting on small sample sizes.

[a]The LA+SA group includes patients who initiated short-acting insulin in the first year; the LA group includes patients who did not initiate short-acting insulin in the first year and who remained on long-acting insulin only.

Abbreviations: CHF, congestive heart failure.; CVA, cerebrovascular accident; CVD, cardiovascular disease; MI, myocardial infarction

©2021 Shroeder EB et al. *JAMA Network Open*.

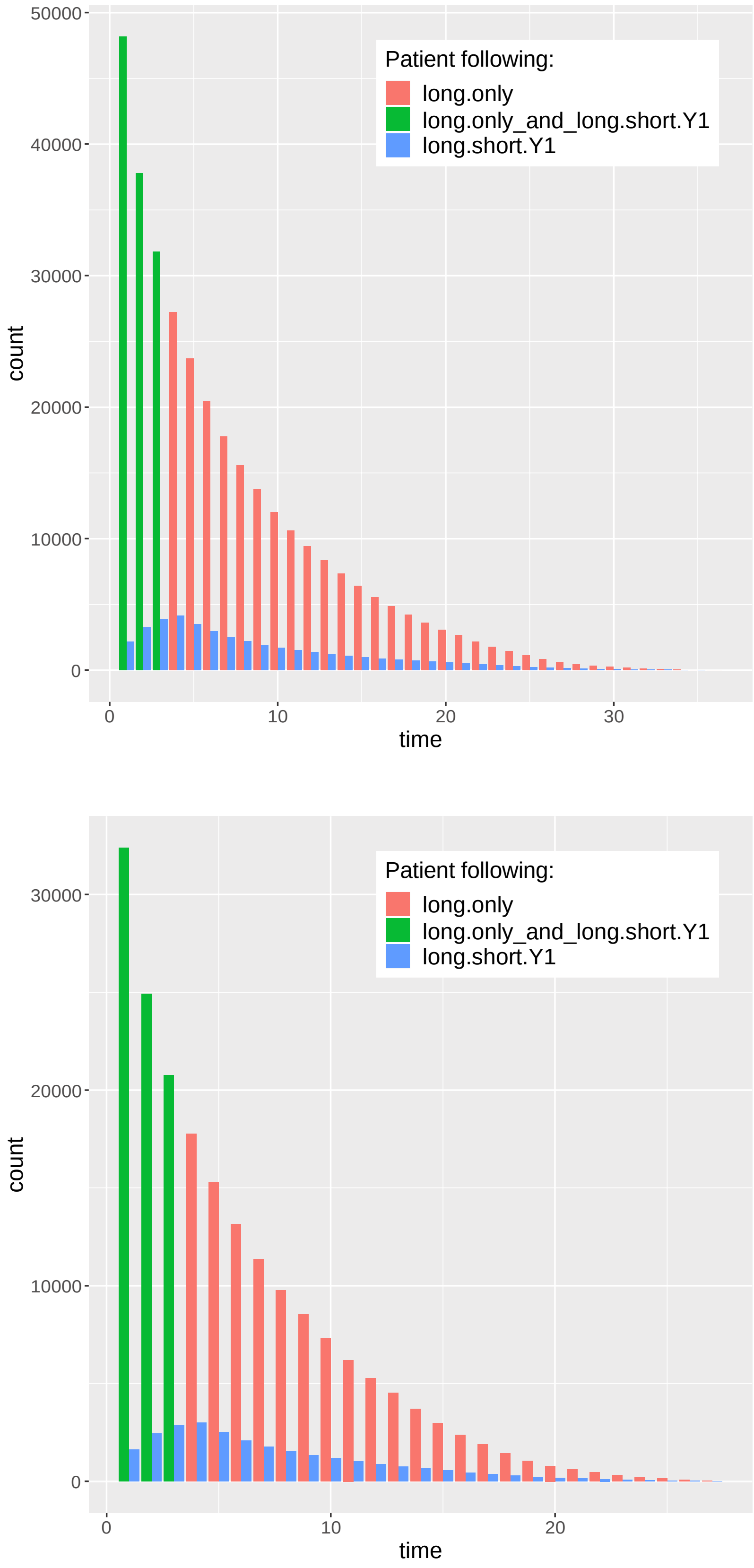

eFigure 1: Data support in the AMI (top panel) and CVD mortality (bottom panel) analyses, i.e., counts of patients following each of the two exposure regimens g_0^* (labeled 'long.only') and g_1^* (labeled 'long.short.Y1') at each quarter of follow-up. The subgroup of patients following both regimens simultaneously is labeled with 'long.only_and_long.short.Y1'.

©2021 Shroeder EB et al. *JAMA Network Open*.

eTable 15: AMI results. The reference exposure regimen is "continuous exposure to long acting insulin only" (i.e., HR>1 or RD>0 means that adding short acting insulin within the first year is deleterious). All results are based on comparing outcomes over 16 quarters (i.e., 4 years). All adjusted results are based on inverse probability weights truncated at 20.

PS estimation approach	HR	AUC	RD1	RD2	RD3	RD4
Crude (no weight)	1.0419 [0.954;1.1297] SE=0.0448, p=0.35	0.286	-3e-04 [-0.0016;9e-04] SE=6e-04, p=0.598	0.0024 [-0.0015;0.0062] SE=0.002, p=0.229	0.0019 [-0.0041;0.0078] SE=0.0031, p=0.54	0.0036 [-0.005;0.0121] SE=0.0044, p=0.412
Logistic 1	0.9085 [0.8009;1.016] SE=0.0549, p=0.095	0.024	-6e-04 [-0.0029;0.0017] SE=0.0012, p=0.619	-0.0057 [-0.0087;-0.0027] SE=0.0015, p=0	-0.005 [-0.0187;0.0088] SE=0.007, p=0.479	-0.006 [-0.0239;0.0118] SE=0.0091, p=0.508
Logistic 2	0.8746 [0.798;0.9512] SE=0.0391, p=0.001	0.015	-1e-04 [-0.0034;0.0031] SE=0.0017, p=0.938	-0.0069 [-0.011;-0.0027] SE=0.0021, p=0.001	-0.0089 [-0.0179;1e-04] SE=0.0046, p=0.052	-0.0149 [-0.0253;-0.0045] SE=0.0053, p=0.005
Logistic 3	0.8869 [0.799;0.9748] SE=0.0449, p=0.012	0.018	-4e-04 [-0.0032;0.0025] SE=0.0014, p=0.799	-0.0067 [-0.0103;-0.003] SE=0.0019, p=0	-0.0076 [-0.0199;0.0047] SE=0.0063, p=0.226	-0.0136 [-0.0268;-4e-04] SE=0.0067, p=0.044
Super Learning	0.886 [0.8068;0.9653] SE=0.0404, p=0.005	0.017	-3e-04 [-0.0032;0.0026] SE=0.0015, p=0.838	-0.0064 [-0.0099;-0.0028] SE=0.0018, p=0	-0.0084 [-0.0196;0.0029] SE=0.0057, p=0.145	-0.014 [-0.0265;-0.0016] SE=0.0064, p=0.027

©2021 Shroeder EB et al. *JAMA Network Open*.

eTable 16: CHF results. The reference exposure regimen is “continuous exposure to long acting insulin only” (i.e., HR>1 or RD>0 means that adding short acting insulin within the first year is deleterious). All results are based on comparing outcomes over 16 quarters (i.e., 4 years). All adjusted results are based on inverse probability weights truncated at 20.

PS estimation approach	HR	AUC	RD1	RD2	RD3	RD4
Crude (no weight)	1.14 [1.0591;1.2208] SE=0.0413, p=0.001	0.019	0.0023 [3e-04;0.0044] SE=0.001, p=0.024	0.0047 [-3e-04;0.0097] SE=0.0025, p=0.063	0.0135 [0.0052;0.0219] SE=0.0042, p=0.001	0.0163 [0.0054;0.0272] SE=0.0056, p=0.003
Logistic 1	1.1118 [0.9353;1.2884] SE=0.0901, p=0.214	0.719	0.002 [-0.0017;0.0056] SE=0.0019, p=0.293	0.0041 [-0.0134;0.0216] SE=0.0089, p=0.646	0.0123 [-0.0102;0.0348] SE=0.0115, p=0.284	0.0263 [-0.0173;0.0699] SE=0.0222, p=0.238
Logistic 2	1.0544 [0.8982;1.2107] SE=0.0797, p=0.495	0.91	7e-04 [-0.0031;0.0045] SE=0.0019, p=0.712	7e-04 [-0.014;0.0154] SE=0.0075, p=0.926	0.012 [-0.0115;0.0355] SE=0.012, p=0.316	0.017 [-0.0144;0.0484] SE=0.016, p=0.288
Logistic 3	1.0725 [0.9079;1.2371] SE=0.084, p=0.388	0.782	0.0017 [-0.0039;0.0074] SE=0.0029, p=0.547	0.0012 [-0.0132;0.0156] SE=0.0073, p=0.87	0.013 [-0.0114;0.0375] SE=0.0125, p=0.296	0.0191 [-0.0149;0.0531] SE=0.0173, p=0.27
Super Learning	1.0714 [0.9053;1.2376] SE=0.0848, p=0.399	0.782	0.0012 [-0.0037;0.0061] SE=0.0025, p=0.628	0.0014 [-0.014;0.0169] SE=0.0079, p=0.854	0.0122 [-0.0124;0.0367] SE=0.0125, p=0.331	0.0202 [-0.0136;0.054] SE=0.0173, p=0.242

©2021 Shroeder EB et al. *JAMA Network Open*.

eTable 17: CVA results. The reference exposure regimen is "continuous exposure to long acting insulin only" (i.e., HR>1 or RD>0 means that adding short acting insulin within the first year is deleterious). All results are based on comparing outcomes over 16 quarters (i.e., 4 years). All adjusted results are based on inverse probability weights truncated at 20.

PS estimation approach	HR	AUC	RD1	RD2	RD3	RD4
Crude (no weight)	1.1152 [0.9856;1.2447]<br>SE=0.0661, p=0.081	0.078	0.0011 [-3e-04;0.0026]<br>SE=7e-04, p=0.127	0.0034 [-2e-04;0.007]<br>SE=0.0018, p=0.066	0.0056 [-2e-04;0.0115]<br>SE=0.003, p=0.06	0.0047 [-0.0026;0.0121]<br>SE=0.0037, p=0.207
Logistic 1	1.0544 [0.6981;1.4108]<br>SE=0.1818, p=0.765	0.918	-4e-04 [-0.0014;5e-04]<br>SE=5e-04, p=0.399	-0.0039 [-0.0064;-0.0015]<br>SE=0.0012, p=0.002	0.0065 [-0.0209;0.0338]<br>SE=0.014, p=0.642	1e-04 [-0.028;0.0282]<br>SE=0.0143, p=0.993
Logistic 2	0.9139 [0.7881;1.0398]<br>SE=0.0642, p=0.18	0.05	-7e-04 [-0.0014;0]<br>SE=3e-04, p=0.049	-0.0044 [-0.0066;-0.0021]<br>SE=0.0011, p=0	-0.0046 [-0.0134;0.0042]<br>SE=0.0045, p=0.301	-0.0096 [-0.0216;0.0025]<br>SE=0.0061, p=0.119
Logistic 3	0.9493 [0.7785;1.1202]<br>SE=0.0872, p=0.561	0.06	-4e-04 [-0.0015;6e-04]<br>SE=5e-04, p=0.402	-0.004 [-0.0065;-0.0016]<br>SE=0.0012, p=0.001	-0.0048 [-0.0129;0.0034]<br>SE=0.0042, p=0.251	-0.0094 [-0.0211;0.0024]<br>SE=0.006, p=0.118
Super Learning	0.938 [0.7896;1.0864]<br>SE=0.0757, p=0.413	0.078	-5e-04 [-0.0014;3e-04]<br>SE=4e-04, p=0.226	-0.0041 [-0.0067;-0.0015]<br>SE=0.0013, p=0.002	-0.0043 [-0.0132;0.0046]<br>SE=0.0045, p=0.342	-0.0101 [-0.0222;0.002]<br>SE=0.0062, p=0.101

©2021 Shroeder EB et al. *JAMA Network Open*.

eTable 18: CVD mortality results. The reference exposure regimen is “continuous exposure to long acting insulin only” (i.e., HR>1 or RD>0 means that adding short acting insulin within the first year is deleterious). All results are based on comparing outcomes over 16 quarters (i.e., 4 years). All adjusted results are based on inverse probability weights truncated at 20.

PS estimation approach	HR	AUC	RD1	RD2	RD3	RD4
Crude (no weight)	1.3634 [1.1608;1.566] SE=0.1034, p=0	0	0.0033 [9e-04;0.0057] SE=0.0012, p=0.008	0.0103 [0.0044;0.0161] SE=0.003, p=0.001	0.0183 [0.0091;0.0275] SE=0.0047, p=0	0.0166 [0.0053;0.028] SE=0.0058, p=0.004
Logistic 1	1.1572 [0.8824;1.432] SE=0.1402, p=0.262	0.613	-3e-04 [-0.0014;9e-04] SE=6e-04, p=0.648	-0.0018 [-0.0059;0.0022] SE=0.002, p=0.371	0.0164 [-0.0117;0.0445] SE=0.0143, p=0.253	0.0121 [-0.0167;0.0408] SE=0.0147, p=0.41
Logistic 2	1.0865 [0.8231;1.35] SE=0.1344, p=0.52	0.702	-8e-04 [-0.0021;5e-04] SE=7e-04, p=0.229	-0.0026 [-0.0078;0.0025] SE=0.0026, p=0.319	0.015 [-0.0171;0.0471] SE=0.0164, p=0.359	0.0097 [-0.0227;0.0421] SE=0.0165, p=0.556
Logistic 3	1.0544 [0.86;1.2489] SE=0.0992, p=0.583	0.767	-8e-04 [-0.0021;5e-04] SE=7e-04, p=0.245	-0.0016 [-0.0081;0.0048] SE=0.0033, p=0.619	0.0067 [-0.0125;0.026] SE=0.0098, p=0.492	0.0013 [-0.0186;0.0211] SE=0.0101, p=0.899
Super Learning	1.0192 [0.8802;1.1582] SE=0.0709, p=0.787	0.901	-9e-04 [-0.0019;1e-04] SE=5e-04, p=0.091	-0.0014 [-0.0078;0.005] SE=0.0033, p=0.676	0.0024 [-0.0127;0.0175] SE=0.0077, p=0.757	-0.004 [-0.0204;0.0123] SE=0.0083, p=0.629

©2021 Shroeder EB et al. *JAMA Network Open*.

eTable 19: All-cause mortality results. The reference exposure regimen is “continuous exposure to long acting insulin only” (i.e., HR>1 or RD>0 means that adding short acting insulin within the first year is deleterious). All results are based on comparing outcomes over 16 quarters (i.e., 4 years). All adjusted results are based on inverse probability weights truncated at 20.

PS estimation approach	HR	AUC	RD1	RD2	RD3	RD4
Crude (no weight)	1.4078 [1.2908;1.5247] SE=0.0597, p=0	0	0.0064 [0.0036;0.0092] SE=0.0014, p=0	0.0215 [0.0146;0.0284] SE=0.0035, p=0	0.0322 [0.022;0.0424] SE=0.0052, p=0	0.0364 [0.0233;0.0495] SE=0.0067, p=0
Logistic 1	1.5872 [1.1925;1.982] SE=0.2014, p=0.004	0.006	0.0115 [-0.0036;0.0265] SE=0.0077, p=0.136	0.0274 [0.0055;0.0492] SE=0.0111, p=0.014	0.0475 [0.0104;0.0845] SE=0.0189, p=0.012	0.0635 [0.0147;0.1123] SE=0.0249, p=0.011
Logistic 2	1.306 [1.0468;1.5652] SE=0.1323, p=0.021	0.041	0.012 [-0.0049;0.0288] SE=0.0086, p=0.164	0.0186 [-9e-04;0.0382] SE=0.01, p=0.062	0.0268 [-0.0017;0.0553] SE=0.0145, p=0.065	0.0407 [0.0018;0.0797] SE=0.0199, p=0.04
Logistic 3	1.2776 [1.0521;1.5032] SE=0.1151, p=0.016	0.033	0.0101 [-0.0041;0.0244] SE=0.0073, p=0.163	0.0185 [2e-04;0.0368] SE=0.0093, p=0.047	0.0216 [-0.0027;0.0458] SE=0.0124, p=0.082	0.0306 [-0.0026;0.0639] SE=0.017, p=0.071
Super Learning	1.2662 [1.0451;1.4872] SE=0.1128, p=0.018	0.037	0.0105 [-0.0042;0.0251] SE=0.0075, p=0.161	0.0183 [1e-04;0.0365] SE=0.0093, p=0.049	0.0202 [-0.0029;0.0433] SE=0.0118, p=0.087	0.0314 [-0.0014;0.0641] SE=0.0167, p=0.06

©2021 Shroeder EB et al. *JAMA Network Open*.

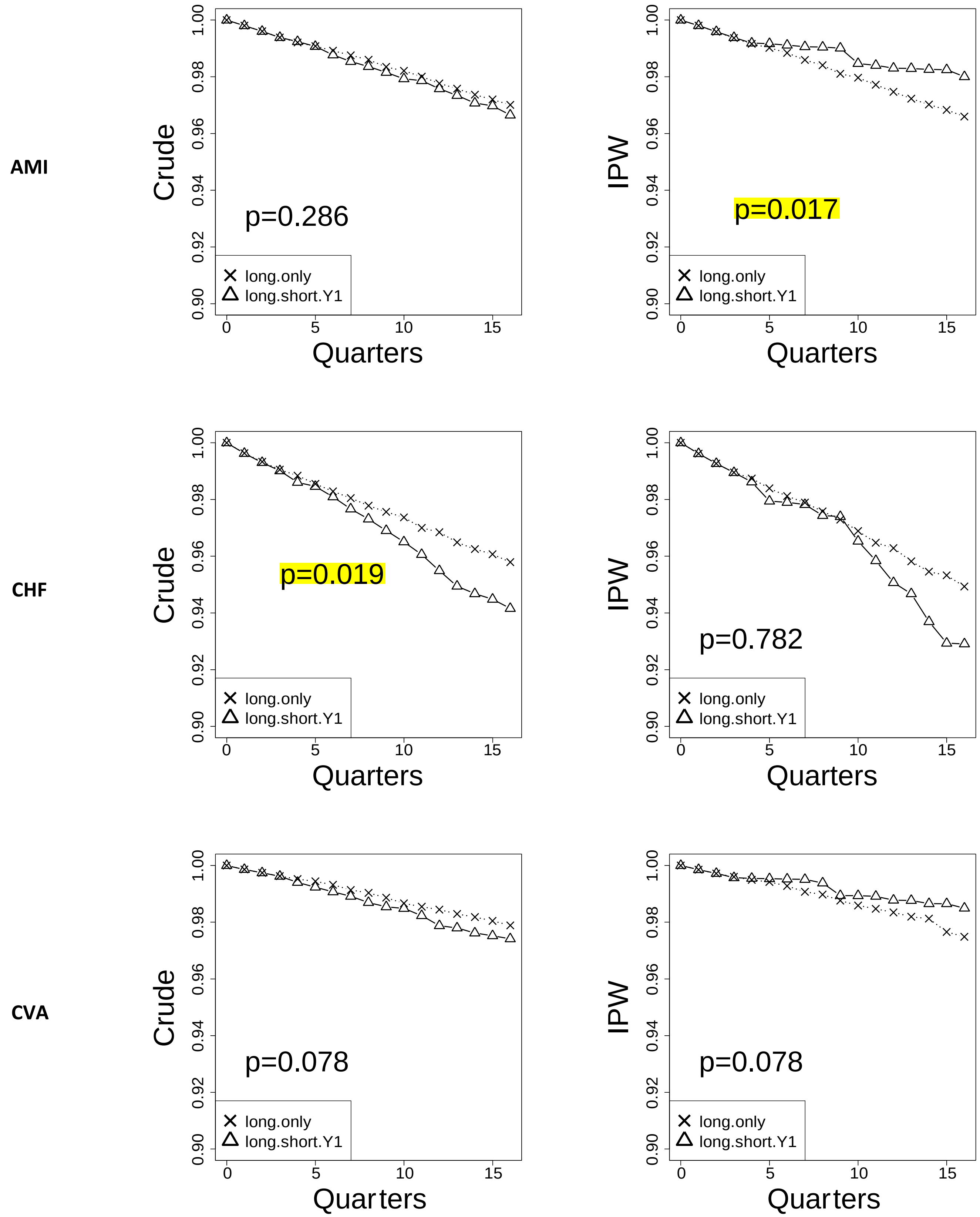

eFigure 2: Survival curve estimates for AMI, CHF, and CVA based on the saturated MSM. The exposure regimens g_0^* and g_1^* are labeled 'long.only' and 'long.short.Y1', respectively. The left plots represent the unadjusted estimates. The right plots represent the truncated IPW estimates based on SL estimation of the propensity scores with truncated IP weights at 20.

©2021 Shroeder EB et al. *JAMA Network Open*.

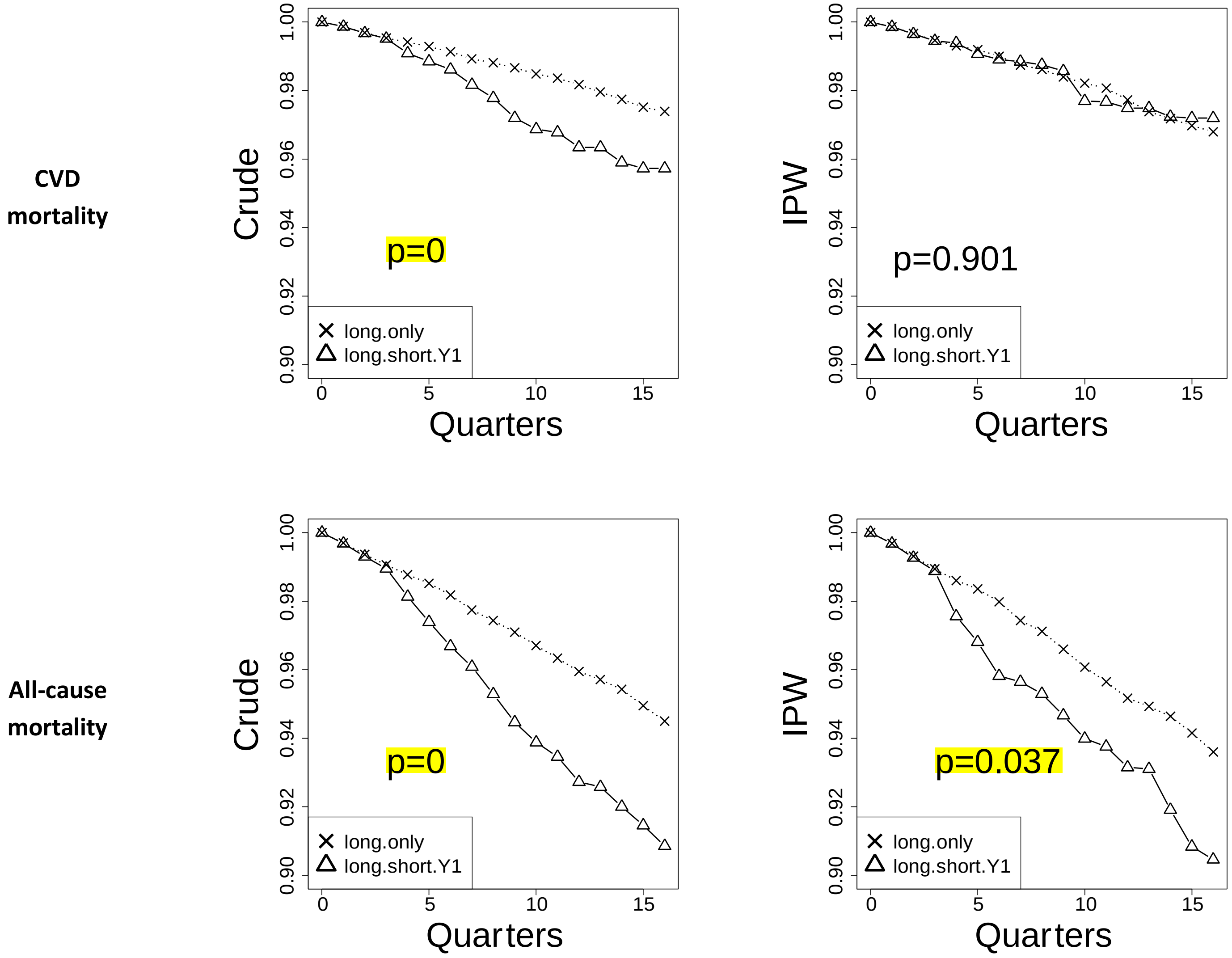

eFigure 3: Survival curve estimates for CVD and all-cause mortality based on the saturated MSM. The exposure regimens g_0^* and g_1^* are labeled 'long.only' and 'long.short.Y1', respectively. The left plots represent the unadjusted estimates. The right plots represent the truncated IPW estimates based on SL estimation of the propensity scores with truncated IP weights at 20.

©2021 Shroeder EB et al. *JAMA Network Open*.

eTable 20: Summary statistics of the inverse probability weights (IPW).

Outcome	PS estimation approach	99th Percentile	99.99^{th} Percentile	Maximum	Percentage of IPW $\geq$ 20
AMI	Logistic 1	2.74	9.97	317.88	0.05
	Logistic 2	3.73	17.34	1205.97	0.08
	Logistic 3	3.83	17.75	1833.76	0.08
	Super Learning	3.14	12.58	1171.04	0.05
CHF	Logistic 1	2.63	9.17	225.22	0.05
	Logistic 2	3.60	14.62	1168.80	0.06
	Logistic 3	3.73	15.28	1023.80	0.06
	Super Learning	3.01	10.84	295.78	0.03
CVA	Logistic 1	2.77	10.27	208.72	0.06
	Logistic 2	3.76	17.46	1192.18	0.08
	Logistic 3	3.87	17.89	1761.55	0.08
	Super Learning	3.16	12.68	1314.70	0.05
CVD mortality	Logistic 1	2.63	8.97	92.40	0.05
	Logistic 2	3.65	14.97	348.88	0.06
	Logistic 3	3.81	15.70	295.01	0.06
	Super Learning	2.08	6.45	103.54	0.01
All-cause mortality	Logistic 1	2.57	8.65	129.21	0.05
	Logistic 2	3.40	13.22	1022.64	0.05
	Logistic 3	3.54	13.89	927.90	0.05
	Super Learning	3.08	10.47	319.11	0.03

©2021 Shroeder EB et al. *JAMA Network Open*.

eReferences

[1] Leong T. K., Tabada G. H., Yang J., Zhu Z., Neugebauer R.. %_MSMstructure SAS macro https://divisionofresearch.kaiserpermanente.org/projects/biostatistics/causalinferencesoftware 2017.

[2] Robins J.M.. Marginal Structural Models in *1997 Proceedings of the American Statistical Association, Section on Bayesian Statistical Science*:1–10 1998.

[3] Cain L. E., Robins J. M., Lanoy E., Logan R., Costagliola D., Hernan M. A.. When to start treatment? A systematic approach to the comparison of dynamic regimes using observational data *Int J Biostat.* 2010;6:Article 18.

[4] Robins J., Orellana L., Rotnitzky A.. Estimation and extrapolation of optimal treatment and testing strategies *Stat Med.* 2008;27:4678–4721.

[5] Neugebauer R., van der Laan M. J.. Nonparametric causal effects based on marginal structural models *Journal of Statistical Planning and Inference.* 2007;137:419 - 434.

[6] van der Laan M. J., Robins J. M.. *Unified methods for censored longitudinal data and causality*. New York: Springer 2003.

[7] Neugebauer R., Schmittdiel J. A., van der Laan M. J.. A Case Study of the Impact of Data-Adaptive Versus Model-Based Estimation of the Propensity Scores on Causal Inferences from Three Inverse Probability Weighting Estimators *Int J Biostat.* 2016;12:131–155.

[8] Polley E.. SuperLearner https://github.com/ecpolley/SuperLearner 2011. Version 2.

[9] Kooperberg C., Bose S., Stone C. J.. Polychotomous Regression *Journal of the American Statistical Association.* 1997;92:117–127.

©2021 Shroeder EB et al. *JAMA Network Open*.
